# Fully Automated Tooth Segmentation and Labeling for Both Full- and Partial-Arch Intraoral Scans Using Deep Learning

**DOI:** 10.1016/j.identj.2025.100950

**Published:** 2025-08-14

**Authors:** Lingyun Cao, Niels van Nistelrooij, Jiaqi Liu, Shankeeth Vinayahalingam, Maximiliano Sergio Cenci, Tong Xi, Bas A.C. Loomans

**Affiliations:** a Department of Dentistry, Research Institute for Medical Innovation, Radboud University Medical Center, Nijmegen, The Netherlands; b Department of Oral and Maxillofacial Surgery, Radboud University Medical Center, Nijmegen, The Netherlands; c School of Stomatology, Xiangyang Polytechnic, Xiangyang, China

**Keywords:** Artificial intelligence, Full-arch intraoral scan, Partial-arch intraoral scan, Tooth segmentation, FDI labeling, Correlation analysis

## Abstract

**Introduction and aims:**

Partial-arch intraoral scans (IOSs) are commonly used in clinical dentistry where high precision and reduced scanning areas are required. However, most existing tooth segmentation algorithms are developed only for full-arch IOSs and perform poorly when applied to partial-arch data. This study aimed to develop a fully automated deep learning (DL) model for tooth segmentation and labeling on both full- and partial-arch IOSs.

**Methods:**

We collected 600 IOSs (300 full-arch and 300 partial-arch) from a dental clinic. The proposed model was based on a two-stage DL model (ToothInstanceNet), and incorporated four enhancements: (1) artificial partial-arch IOSs, (2) DL-based alignment module, (3) FDI-aware postprocessing algorithm, and (4) real partial-arch IOSs. Model performance was evaluated via 5-fold cross-validation using F1-score, tooth Dice, tooth macro-F1, and macro-IoU. In addition, the model was evaluated with the public Teeth3DS dataset, and we analysed correlations between dental conditions and model errors.

**Results:**

The model achieved an F1-score of 0.9908 and 0.9884; tooth Dice of 0.9819 and 0.9862; tooth macro-F1 of 0.9940 and 0.9786; and macro-IoU of 0.9403 and 0.9280 on full- and partial-arch IOSs, respectively. The model also demonstrated superior performance (score = 0.9870) in 3DTeethSeg challenge. Correlation analyses revealed that certain dental conditions, particularly residual roots, residual crowns, missing teeth, and partially erupted teeth, were significantly and positively associated with the model’s errors.

**Conclusions:**

The current study proposes the first fully automated method for tooth segmentation and FDI labeling on both full- and partial-arch IOSs. The final model demonstrated high accuracy for both scan types, indicating its potential for integration into clinical dental work.

**Clinical relevance:**

This work could aid clinicians in the first step of tooth identification in digital dental workflows, and lays the groundwork for extending the automation of the downstream applications, such as diagnosis and monitoring on partial-arch IOSs.

## Introduction

In contemporary digital dental workflows, intraoral scans (IOSs) play an important role throughout diagnosis, treatment planning, and monitoring.^1^, ^2^, ^3^ By projecting structured light or a laser onto teeth and surrounding structures, IOS devices capture and generate high-resolution three-dimensional (3D) representations of a patient’s dental anatomy.^4^ With advantages such as efficiency, patient comfort, cost-effectiveness, user-friendliness, and ease of storage, IOSs have increasingly replaced traditional impression techniques in clinical practice.^5^ Today, applications of IOSs can be found across various fields, including orthodontics, prosthodontics, implantology, and maxillofacial surgeries.^6^, ^7^, ^8^

An IOS can be categorized as full-arch or partial-arch based on the extent of coverage. Full-arch IOSs cover the entire upper or lower dental arch, while partial-arch IOSs focus on specific regions, such as a quadrant or sextant.^9^^,^^10^ When IOS technology was first introduced, it was primarily limited to partial-arch scans due to cumulative stitching errors that distort disparate scan areas.^11^ This restriction was later overcome with advances in scanning technology, leading to the development of full-arch IOSs.^11^

Nevertheless, studies have reported that today partial-arch IOSs still provide higher trueness and precision compared to full-arch IOSs for both anterior and posterior regions.^10^^,^^12^^,^^13^ This could be attributed to the smaller scanning area, which decreases the likelihood of errors and distortions when stitching the captured images into a 3D mesh.^9^ Partial-arch IOSs are commonly employed to generate digital designs for crowns, inlays, and bridges, offering accurate delineation of prepared teeth. Current consensus recommends using partial-arch IOSs when a limited scanning area is required, as they provide several clinical advantages, including reduced scanning time, enhanced patient comfort, and higher precision.^12^^,^^13^

Artificial intelligence (AI) refers to the ability of computers to mimic human intelligence and perform complex tasks.^14^^,^^15^ Deep learning (DL), an advanced subset of AI, is distinguished by its capacity to autonomously learn features from input data and generate outputs without explicit programming.^14^, ^15^, ^16^ By identifying intrinsic statistical patterns and structures, DL enhances reliability and streamlines repetitive tasks.^17^ In dentistry, DL has been applied to automate various tasks related to IOSs, including tooth segmentation, measurement of tooth wear, integration with cone-beam computed tomography, and the design of dental restorations.^6^^,^^18^, ^19^, ^20^

However, current DL-related studies are mainly focused on full-arch IOSs. It has been reported that existing high-performing algorithms developed for full-arch IOSs underperformed significantly when applied to partial-arch IOSs.^21^ Our study aimed to develop a fully automated method for tooth segmentation and labeling on both full- and partial-arch IOSs collected from real-world clinical settings. This research represents a foundational step for expanding AI-driven automation into underexplored partial-arch scenarios and supports future applications in digital workflow integration across diverse scanning formats.

## Materials and methods

This study followed the ethical principles of the World Medical Association (Declaration of Helsinki).^22^ Informed consent was collected from each patient, and all patient data was pseudonymized before analysis. A data transfer agreement was secured (Radboudumc File #A24-2297), and ethical approval for the use of patient data has been granted by the institutional review board (METC Oost-Nederland, file number 2024-17756). The checklist for AI research in dentistry was consulted to prepare this manuscript.^23^

### Data collection

A total of 600 IOSs from 300 patients acquired between January 2021 and June 2024 in a private dental clinic in Hubei, China, were retrospectively included in this study, including 300 full-arch IOSs and 300 partial-arch IOSs. All scans were acquired using the 3Shape Trios Move or the 3Shape D500 (3shape) following the manufacturer’s guidelines and exported in PLY format without colour information.

The definitions for full-arch IOS and partial-arch IOS were as follows:

1. Full-arch IOS: Continuous scan of the entire maxillary or mandibular dental arch with the two first molars thoroughly presented (or the gingiva of the corresponding site for those with lost first molars).
2. Partial-arch IOS: All other scans were categorized as partial-arch IOSs.

**Fig. 1:**
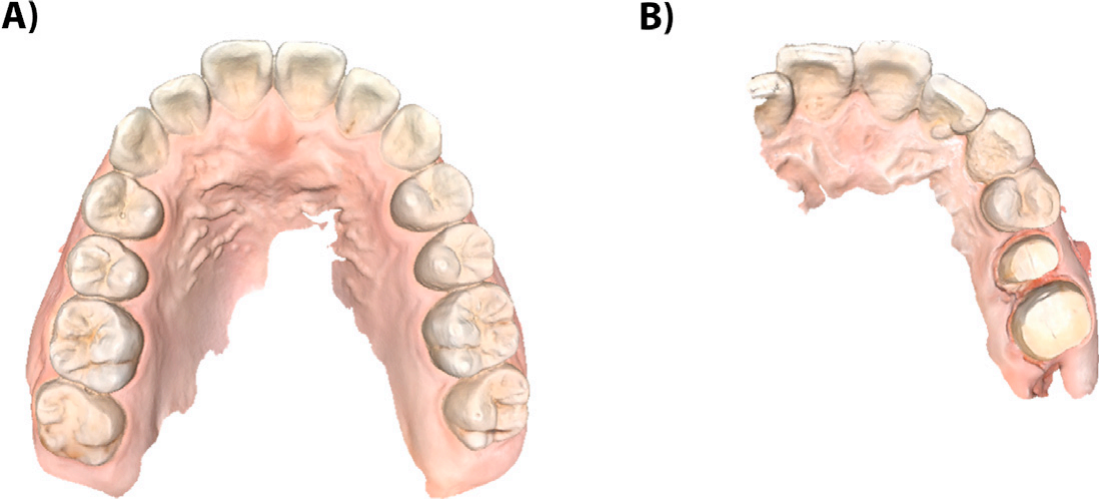
Representative examples of intraoral scans (IOSs). Part (A) shows a full-arch IOS capturing the entire dental arch. Part (B) shows a partial-arch IOS focused on a localized region.

Figure 1 presents examples of a full-arch IOS and a partial-arch IOS.

The inclusion and exclusion criteria were as follows:

• Inclusion criteria:

1. Paired upper and lower scans from the same patient collected for treatment planning and monitoring purposes.
2. Patients with diverse treatment needs and dental conditions were included, such as those with missing teeth, prepared teeth, implants, orthodontic appliances, residual roots, residual crowns, and partially erupted teeth.

• Exclusion criteria:

1. Patients with primary or mixed dentitions.
2. Patients without a paired upper and lower IOS, or with both a full-arch and partial-arch IOS.
3. Patients with poor-quality IOSs due to low resolution or inaccurate stitching.
4. Patients with IOSs involving teeth/prosthetics that cannot be numbered according to the FDI (Fédération Dentaire Internationale, World Dental Federation) notation system.^24^
5. Duplicate or longitudinal follow-up scans from the same patient.

### Data annotation

Teeth were annotated using point-wise segmentations and labelled according to the FDI notation system with the 3DMedX software (3DLab). Artificial prosthetics were assigned to their corresponding FDI labels, including implants and crowns (single crowns, bridges, and dentures), residual roots, residual crowns, and visible partially erupted teeth. Teeth that were incompletely scanned were included, and any disconnected surface patches were interpreted as one tooth.

The segmentation and annotation processes were performed twice by a dentist (LC) with 5 years of experience, with a 3-month interval between the two sessions. Discrepancies between the two segmentations were reviewed by a PhD candidate (NvN) with good experience on tooth annotation, and the final annotations were determined through discussions between LC and NvN.

In addition, for each partial-arch IOS, the number of scanned teeth, the count of specific dental conditions (including missing teeth, prepared teeth, implants, orthodontic appliances, residual roots, residual crowns, and partially erupted teeth), as well as the total count of dental conditions per scan, was recorded.

### DL model

To improve performance on partial-arch IOSs, the proposed model was built upon ToothInstanceNet^25^ by incorporating four major enhancements: (1) artificial partial-arch IOSs as data augmentation, (2) prealignment of the IOS to a standard orientation, (3) incorporating the structure of the FDI notation system into the postprocessing, and (4) real partial-arch IOSs during training.

#### Established method

ToothInstanceNet^25^ used two stages that process a low-resolution and high-resolution point cloud from the IOS, respectively. Stage 1 predicts tooth instance segmentations and FDI labels inspired from DentalNet,^26^ and stage 2 refines the segmentations based on small crops of individual teeth. ToothInstanceNet combines the large-context predictions of tooth labels with the high-resolution predictions of tooth segmentations.

#### Artificial partial-arch IOSs

A strong data augmentation technique to train an effective model for partial-arch IOSs using only full-arch IOSs would be to crop convincing partial-arch scans from the full-arch scans.^27^ This was done as follows:

1. Determine a sequence of teeth in the dental arch;
2. Randomly select 2 to 12 consecutive teeth;
3. Crop by removing surface area outside of arch segment of selected teeth;
4. Translate the crop to centre it at the origin.

The sequence was determined by following the FDI labels from left to right. To remove the surface area, an oriented bounding box was determined around the selected teeth, and only the largest connected surface inside the box was kept. To increase the data diversity, this data augmentation was applied with a 90% probability, and the random number of selected teeth was sampled from a skewed distribution with higher probabilities for fewer teeth.

#### Prealignment

ToothInstanceNet used principal component analysis to establish a rough orientation of a full-arch scan.^28^ This statistical method is not effective for partial-arch scans, as tooth surfaces required to determine the correct pose are missing. Therefore, the current study proposes a DL method for aligning full-arch and partial-arch IOSs to a standard orientation inspired from.^29^

A total of 234 full-arch IOSs with two first molars and two central incisors were eligible for the development of this stage. First, the occlusal plane was determined by the two centroids of the first molars and the mean centroid of the central incisors. Each IOS was centred on the origin, and the anterior-posterior direction was made to follow the y-axis ($d_{forward}=\left[0,-1,0\right]$) by aligning the centroids of the first molars in the x-direction and aligning the normal vector of the occlusal plane in the coronal direction with the z-axis ($d_{up}=\left[0,0,1\right]$). The IOSs of the upper and lower dental arch were thus oriented the same way.

A standardized IOS was randomly rotated to establish a new orientation, and the principal component analysis method for pose normalization was used to roughly align the rotated IOS. The modified directions ($d{{}^{\prime }}_{forward}\in R^{3}$, $d{{}^{\prime }}_{up}\in R^{3}$) were directly predicted by the model (${\hat{d}}_{forward}$, ${\hat{d}}_{up}$) and supervised according to:

$$L_{orient}=2-\frac{{d^{\prime }}_{forward}\cdot {\hat{d}}_{forward}}{\left|{\hat{d}}_{forward}\right|}-\frac{{d^{\prime }}_{up}\cdot {\hat{d}}_{up}}{\left|{\hat{d}}_{up}\right|}+\left|\frac{{\hat{d}}_{forward}\cdot {\hat{d}}_{up}}{\left|{\hat{d}}_{forward}\right|\left|{\hat{d}}_{up}\right|}\right|,$$

$L_{orient}$ optimizes the cosine angles between the modified and predicted directions and ensures orthogonality.

In order to align a partial-arch scan of posterior elements to a standard orientation, it needs to be translated either to the left or right. A ground-truth translation vector ($c{}^{\prime }\in R^{3}$) can be acquired at step d) of partial-arch IOS generation. Therefore, the model additionally predicted this translation vector ($\hat{c}$), which was supervised using the smooth-L1 loss function. Furthermore, to provide some auxiliary supervision, the model also predicted a point-level binary segmentation of teeth ($s{}^{\prime }\in R^{N}$), which was compared against the ground-truth segmentation ($\hat{s}$) using the binary cross-entropy (BCE) and Dice loss functions. In summary, the alignment loss function is as follows:

$$L_{align}=L_{orient}+\text{Smooth}-L1\left(c^{\prime },\hat{c}\right)+\text{BCE}\left(s^{\prime },\hat{s}\right)+\text{Dice}\left(s^{\prime },\hat{s}\right)$$

#### FDI numbering postprocessing

The predictions of tooth numbers were converted back to FDI labels using a novel postprocessing method based on class probabilities and expected tooth pair offsets. The FDI numbers of either the lower or upper arch were determined.

1. ***Sequence of teeth***: After aligning an IOS, the most posterior tooth was selected as initial tooth, and a sequence of teeth following the dental arch was determined by iteratively including a tooth according to the minimum cosine angle to the last included tooth. The resulting sequence could go from left to right or vice versa.
2. ***Tooth number costs***: The class logits of each tooth were transformed to a probability distribution using the softmax function and converted to costs as negative log values.
3. ***Tooth pair costs***: Based on the aligned scans and annotations, a set of 3D offsets was collected for every pair of FDI numbers:
  $$o_{k}^{\left[i,\;j\right]}\in R^{3}\left|i\in \left\{11,\ldots ,48\right\},\;j\;\in \left\{11,\ldots ,48\right\},\;k=1,\ldots ,\;k\right\}c$$
  The offsets were computed as the difference in centroids of two teeth corresponding to the FDI number pair. Offsets of one FDI pair were copied to the reflected FDI numbers (eg, 21-16 to 11-26) to increase the number of offsets per pair. Furthermore, connected components were determined for each annotated tooth, and the offsets were computed based on their centroids, to be able to collect offsets between centroids with the same FDI number. Every FDI pair with the same numbers within an arch was given all the same-FDI offsets of that arch to increase the number of offsets.
  Each set of offsets was modelled using a multivariate Gaussian distribution ($p\left(o^{\left[i,j\right]}\right)∼N\left(\mu ,\Sigma \right)$) with mean vector μ and covariance matrix Σ. When postprocessing a new IOS, the probability densities of the offsets between subsequent teeth in the sequence were computed for all possible FDI number pairs (16 × 16 per arch) and converted to tooth pair costs as negative log values.
4. ***Minimum-cost FDI sequence:*** The optimal sequence of FDI numbers was determined as the sequence with the minimum cost by summing the tooth number costs and tooth pair costs along the sequence. A dynamic programming algorithm was developed to determine the optimal sequence in polynomial time. This algorithm started with the costs of attributing each FDI number to the first tooth, which were given by the tooth number costs. The algorithm iteratively found the minimum cost of attributing every FDI number up to the next tooth until the minimum costs up to the final tooth were computed. The minimum-cost predecessors were recorded at each iteration to recover the minimum-cost sequence after running the algorithm.

#### Real partial-arch IOSs

Real partial-arch IOSs were further added to the training pipeline to make the model more adaptable to real-world clinical settings.

### Model training

The alignment model was implemented using the Stratified Transformer architecture^30^ based on Pytorch Lightning (v. 2.3.3).^31^^,^^32^ Global average pooling and a multilayer perceptron were applied to the point features at the most latent dimension to predict the alignment parameters (d̂_forward, d̂_up, ĉ). A batch size of 8 and a base learning rate of 0.001 were used for a total of 1000 epochs. Other training parameters are taken over from our previous study.^25^

### Model inference

An IOS was prepreprocessed to a low-resolution point cloud, and the alignment parameters were determined by the alignment model. The predicted translation was applied in reverse to translate the IOS to a standard position. Then, the forward direction was made orthogonal to the up direction, and the left-right direction was determined by the cross product between the forward and up directions. These three directions were used as an orthonormal basis to rotate the translated IOS to a standard orientation.

The subsequent stages for tooth instance segmentation and single-tooth binary segmentation were applied to the standardized IOS. Test-time augmentation in the form of horizontal flipping was applied to the instance stage to increase sensitivity, and the single-tooth stage was run a second time with updated tooth centroids to improve the segmentation of approximal surfaces. The final tooth segmentation was postprocessed by a graph-cut algorithm, and the tooth number predictions were postprocessed to FDI numbers using the method described previously.

### Model setting

**Fig. 2:**
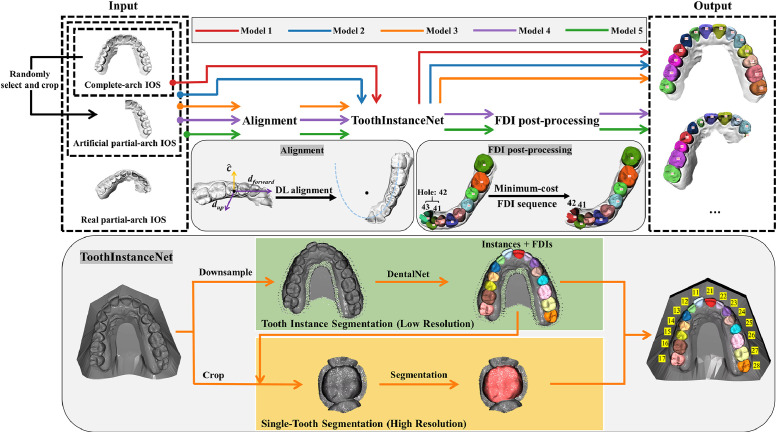
Overview of the proposed deep learning (DL) pipeline for tooth segmentation and FDI labeling. Three types of intraoral scans (IOSs) – full-arch, artificially cropped partial-arch, and real partial-arch – were used as model inputs. Five models were developed, marked in different colours: Model 1 (default model, ToothInstanceNet), Model 2 (added artificial partial-arch IOSs), Model 3 (added alignment module), Model 4 (added FDI-aware postprocessing), and Model 5 (added real partial-arch IOSs). The alignment module predicts up and forward directions and a translation vector to standardize scan orientation. The FDI postprocessing module assigns optimal tooth labels based on class probabilities and expected tooth pair offsets. ToothInstanceNet performs two-stage segmentation, combining a low-resolution instance segmentation via DentalNet^26^ and high-resolution single-tooth refinement.

The alignment model was always trained on the full-arch scans with both first molars and both central incisors. The other stages were trained with the following configurations (Figure 2):

1. Model 1: default;
2. Model 2: default + artificial partial-arch IOSs;
3. Model 3: default + artificial partial-arch IOSs + alignment;
4. Model 4: default + artificial partial-arch IOSs + alignment + postprocessing;
5. Model 5: default + artificial partial-arch IOSs + real partial-arch IOSs + alignment + postprocessing.

### Statistical analysis

The full method and the proposed components were evaluated using 5-fold cross-validation.^33^ Statistical analyses were performed using scikit-learn (v. 1.5.1).^34^

Tooth detection was evaluated by matching predicted and annotated teeth with a point-wise intersection over union (IoU) = $\frac{TP}{TP+FP+FN}$ of at least 0.5 and computing tooth-wise F1-score (ie, Dice score) = $\frac{2\times TP}{2\times TP+FP+FN}$, where TP, FP, and FN are true-positive, false-positive, and false-negative detections, respectively. Tooth segmentation was investigated by the point-wise Dice score between matched teeth, and tooth labeling was evaluated with macro-F1 for matched teeth. The overall effectiveness was evaluated using macro-IoU, which averages the point-wise IoU values of each FDI label.

In addition, the publicly available Teeth3DS dataset^28^ for 3DTeethSeg challenge^35^ was also utilized in this study, comprising 1800 annotated full-arch IOSs from 900 patients with lower and upper scans. The 3DTeethSeg challenge evaluated tooth detection with the teeth localization accuracy (TLA), defined as the mean of tooth-size-normalized Euclidean distances between annotated teeth and the closest predicted tooth. Furthermore, it defined teeth segmentation accuracy (TSA) as the F1-score comparing all annotated and predicted tooth points. Lastly, tooth labeling was evaluated using tooth identification rate (TIR), which was defined as the percentage of annotated teeth with a closely predicted tooth labelled with the same FDI label. Overall effectiveness was evaluated by averaging these metrics.

Correlations between dental conditions of partial-arch IOSs and false positives, false negatives, and wrong labels of Model 5 were analysed by calculating Kendall’s tau-b correlation coefficients (*τ*) and *P* values, with a *P* value lower than .05 considered statistically significant.

## Results

### Description of datasets

The dataset collected from dental clinic presents a wide variance of dental conditions. Table 1 summarizes the count and proportion of scans with missing teeth, prepared teeth, implants, orthodontic appliances, residual roots, residual crowns, and partially erupted teeth. Notable differences could be observed between full- and partial-arch IOSs. For example, the most common dental condition for full-arch IOSs is missing teeth (35.67%), whereas in partial-arch scans, prepared teeth are most frequently observed (34.33%). Notably, our real-world data shows 54.67% of all IOSs contained at least one dental condition.

**Table 1:** Counts and proportions of the scans with specific dental conditions in 300 full-arch IOSs and 300 partial-arch IOSs.

Types	Full-arch IOS count (%)	Partial-arch IOS count (%)	Total count (%)
**Missing tooth**	**107 (35.67)**	91 (30.33)	**198 (33.00)**
**Prepared tooth**	43 (14.33)	**103 (34.33)**	146 (24.33)
**Implant**	5 (1.67)	2 (0.67)	7 (1.17)
**Orthodontic appliance**	13 (4.33)	3 (1.00)	16 (2.67)
**Residual root**	19 (6.33)	12 (4.00)	31 (5.17)
**Residual crown**	7 (2.33)	6 (2.00)	13 (2.17)
**Partially erupted tooth**	20 (6.67)	14 (4.67)	34 (5.67)
**Scans with ≥1 dental condition (s)**	141 (47.00)	187 (62.33)	328 (54.67)

Most commonly observed dental conditions in each type IOS are highlighted bold.

IOS, intraoral scan.

**Fig. 3:**
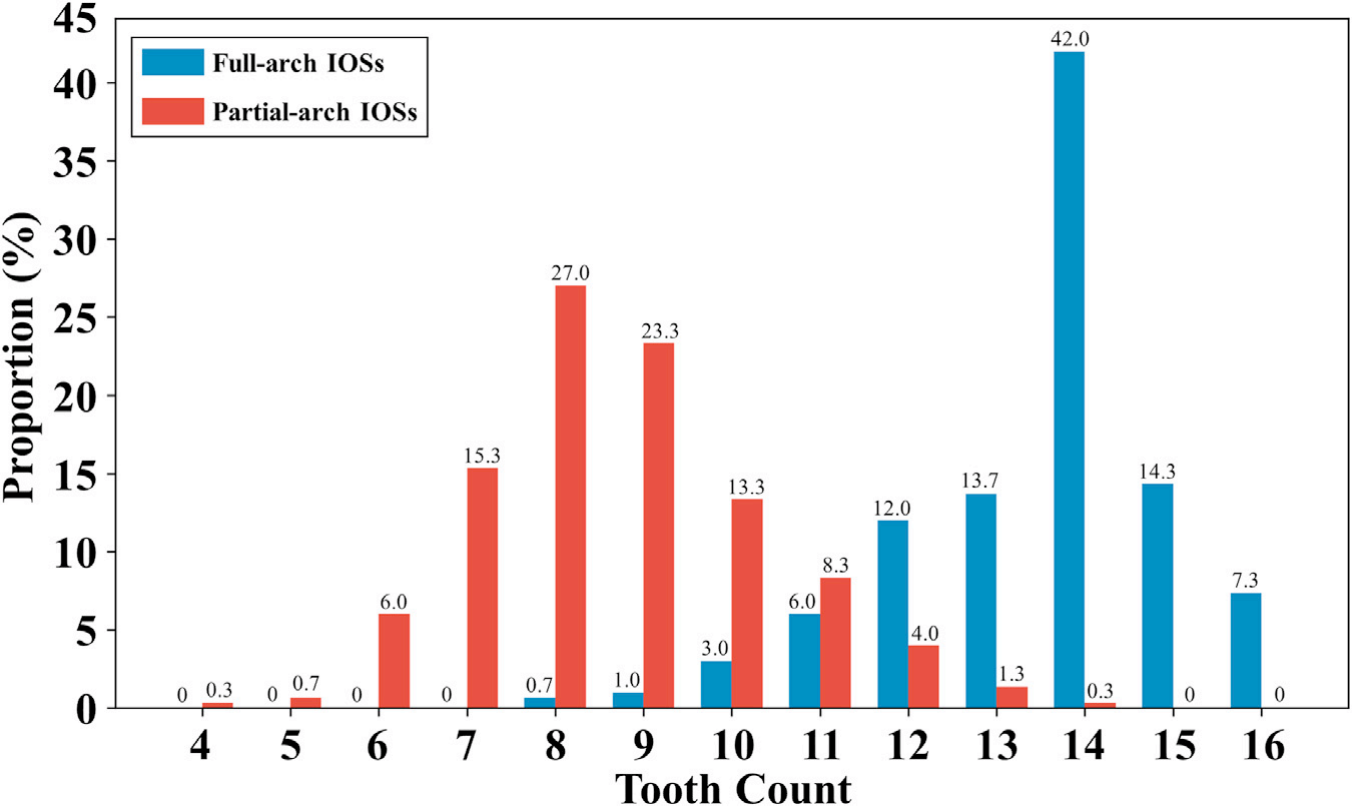
Distribution of tooth counts per arch in 300 full-arch and 300 partial-arch intraoral scans (IOSs).

The distribution of tooth counts per arch is presented in Figure 3. The most frequently observed tooth count in partial-arch IOSs is 8, whereas for full-arch IOSs, the most common count is 14.

### Quantitative results of the ablation study

The results of the model configurations on our dataset are presented in Table 2. For full-arch IOSs, all five models (Model 1-5) demonstrated high effectiveness in tooth detection (F1-score), tooth segmentation (tooth Dice), and tooth labeling (tooth macro-F1). Model 5 outperformed the other configurations in most metrics, whereas Model 1 demonstrated the highest F1-score. Model 5 achieved the fewest wrong labels (18), while Model 1 exhibited the fewest false positives (25), false negatives (21), and wrong cases (52). For partial-arch IOSs, Model 5 achieved the best overall performance across all metrics. Although Model 3 had the fewest false positives (37), Model 5 had the fewest false negatives (23), wrong labels (56), and wrong cases (64). Based on these results, Model 5 was selected as the best final model for this study.

**Table 2:** Results of ablation study for the deep learning (DL) models on the collected datasets (both full-arch and partial-arch).

Dataset	Method	F1-score	Tooth Dice	Tooth macro-F1	Macro-IoU	FP	FN	Wrong label	Wrong cases
**Full-arch IOS**	Model 1	**0.9935**	0.9816	0.9893	0.9331	**25**	**21**	30	**52**
	Model 2	0.9911	0.9806	0.9788	0.9053	27	32	61	73
	Model 3	0.9899	0.9818	0.9908	0.9350	38	29	28	61
	Model 4	0.9902	0.9818	0.9926	0.9361	37	28	20	53
	Model 5	0.9908	**0.9819**	**0.9940**	**0.9403**	36	25	**18**	57
**Partial-arch IOS**	Model 1	0.9751	0.9810	0.7823	0.6537	45	83	639	256
	Model 2	0.9828	0.9812	0.9336	0.8487	39	52	170	150
	Model 3	0.9865	0.9856	0.9452	0.8724	**37**	34	149	113
	Model 4	0.9865	0.9857	0.9619	0.8995	38	34	110	80
	Model 5	**0.9884**	**0.9862**	**0.9786**	**0.9280**	38	**23**	**56**	**64**

Best metrics are highlighted bold. F1-score, tooth Dice, tooth macro-F1, macro-IoU reflect the model’s performance in tooth detection, segmentation, labeling, and overall effectiveness, respectively. False positive, false negative, and wrong label represent the cumulative tooth-level error counts across all scans; wrong cases refer to the number of scans containing at least one error.

FN, false negative; FP, false positive; IOS, intraoral scan.

### Model performance on the public dataset

On the public Teeth3DS dataset, our model achieved a precision of 0.9933, sensitivity of 0.9994, F1-score of 0.9963, tooth Dice of 0.9823, tooth macro-F1 of 0.9676, and macro-IoU of 0.9029.

Additionally, our final model outperformed state-of-the-art methods reported by Rekik et al^36^ across all key metrics in the 3DTeethSeg challenge, including TLA, TSA, TIR, and overall score (Table 3).

**Table 3:** Results of our final model and comparative state-of-the-art methods on public Teeth3DS dataset, reported based on the 3DTeethSeg challenge metrics.

Model	TLA	TSA	TIR	Score
**TSegNet**	0.9521	0.9686	0.9295	0.9501
**MeshSegNet**	0.9334	0.8702	0.8319	0.8785
**RHL**	0.9734	0.9707	0.9567	0.9669
**DTSegNet**	0.9408	0.9437	0.9354	0.9340
**TSegLab**	0.9845	0.9817	0.9761	0.9808
**Our Model**	**0.9945**	**0.9862**	**0.9803**	**0.9870**

Most effective metrics are highlighted bold. Score = (TLA + TSA + TIR)/3. Results of TSegNet, MeshSegNet, RHL, DTSegNet, and TSegLab were reported by Ahmed Rekik et al.^36^

TIR, teeth identification rate; TLA, teeth localization accuracy; TSA, teeth segmentation accuracy.

### Qualitative analysis

**Fig. 4:**
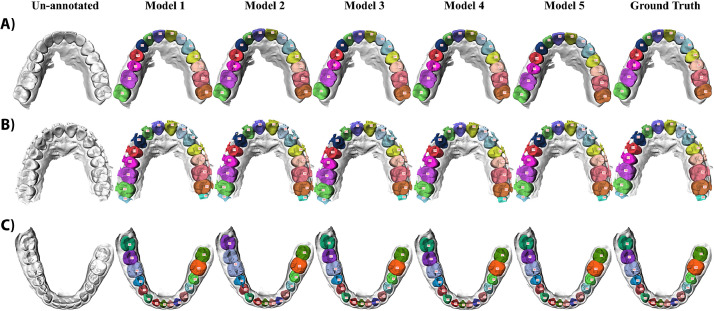
Qualitative results for full-arch IOSs. Parts (A-C) provide three representative examples of full-arch IOSs with bridge, orthodontic appliance, and wisdom tooth, respectively. The first column shows the input scan, columns two to six show the predictions of Models 1 to 5, and the last column shows the reference annotations (ground truth). Teeth are labelled using the FDI notation system.

**Fig. 5:**
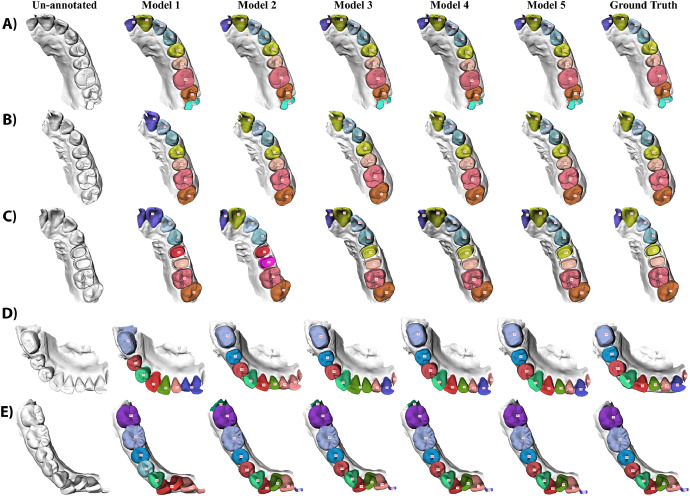
Qualitative results for partial-arch IOSs. Parts (A-E) provide five representative examples of partial-arch IOSs, illustrating the progressive improvement from Models 2 to 5. The first column shows the input scan, columns two to six show the predictions of Models 1 to 5, and the last column shows the reference annotations (ground truth). Teeth are labelled using the FDI notation system.

The qualitative results further demonstrated the performance of the proposed method in handling complex dental conditions. Figures 4 and 5 present the performance of the five models on three full-arch IOSs and five partial-arch IOSs, respectively.

For full-arch IOSs, Figure 4A shows a full upper arch with a dental bridge covering teeth 13 to 22. Figure 4B shows a full upper arch with fixed orthodontic appliance. All five models produced accurate results in these two cases. Figure 4C shows a full lower arch with teeth from 37 to 48. Model 2 incorrectly labelled teeth 46 and 47 both as 46, labelled 48 as 47, and overannotated 45; whereas all other models produced correct results in this case.

For partial-arch IOSs, Figure 5A shows a partial upper arch where all five models produced accurate results. Figure 5B shows a partial upper arch improved by Model 2. Model 1 mislabelled tooth 21 as 11. Models 2 to 5 all delivered accurate results. Figure 5C shows a partial upper arch improved by Model 3, with tooth 24 and 25 as prepared teeth. Model 1 misclassified 11 and 21 as a single 11, and labelled 24 as 14; Model 2 labelled 24 and 25 as 14 and 15; Models 3 to 5 produced accurate results. Figure 5D shows a partial lower arch improved by Model 4, with tooth 46 as a prepared tooth. Model 1 overannotated tooth 46, incorrectly labelled teeth 41 to 45 as 31 to 44, labelled 31 and 32 both as 32, and missed 33 at the scan margin; Model 2 labelled 32 and 33 both as 33; Model 3 labelled 41 and 42 both as 41; Model 4 and Model 5 produced accurate results. Figure 5E shows a partial lower arch improved by Model 5. Model 1 labelled 44 as 34, 41 and 42 both as 42, and 31 and 32 both as 31; Model 2, Model 3, and Model 4 detected 48, which was not present; only Model 5 produced accurate results.

### Failure cases

**Fig. 6:**
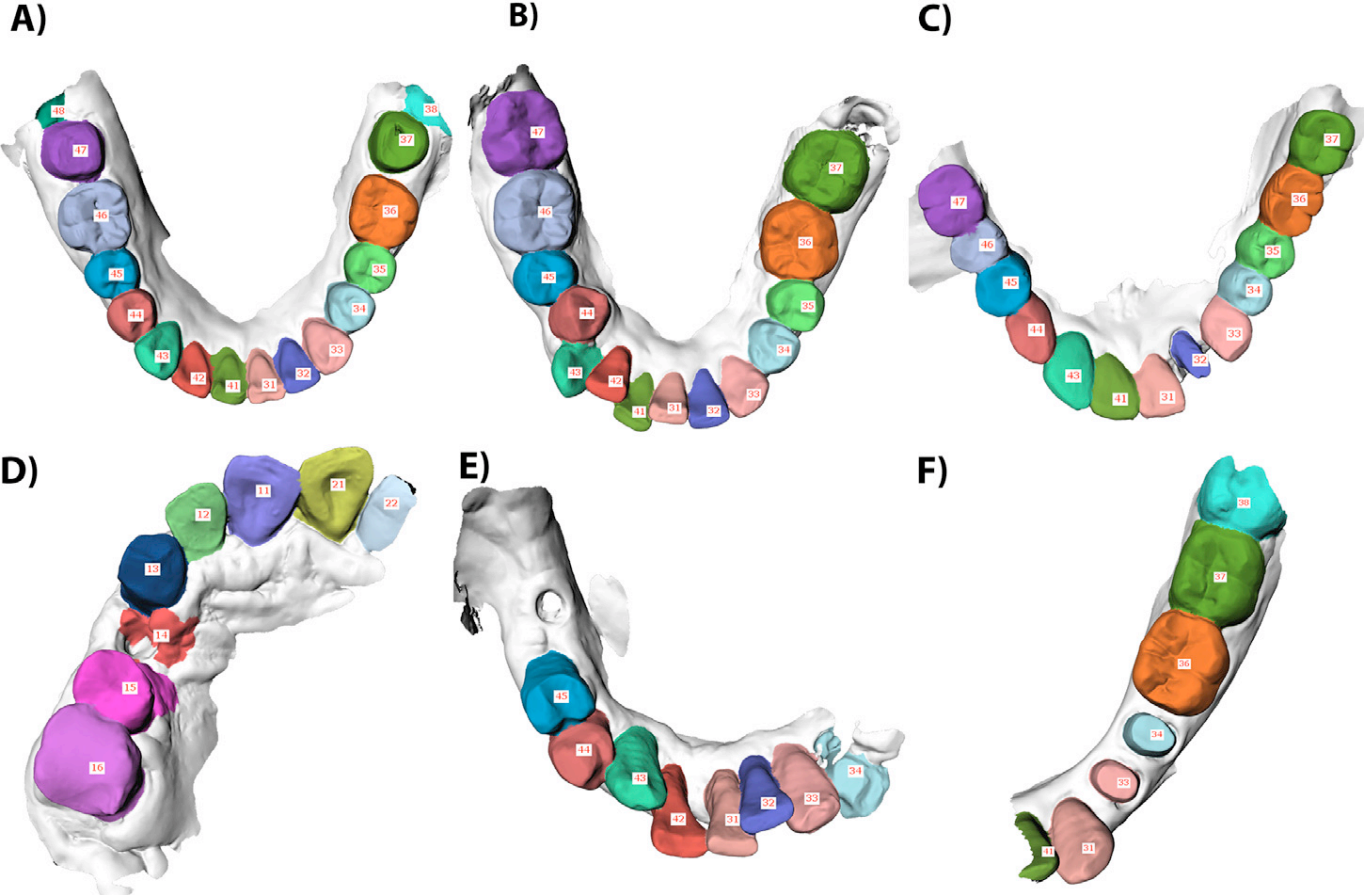
Failure cases from Model 5 of false positive, false negative, and wrong labels on full-arch IOSs and partial-arch IOSs. Parts (A-C) provide three failure cases on full-arch IOSs. Parts (D-F) provide three failure cases on partial-arch IOSs.

Examples of six failure cases are presented in Figure 6, showing the false positive, false negative, and wrong labels on full-arch IOSs and partial-arch IOSs.

For full-arch IOSs, Figure 6A shows a full lower arch with two prepared teeth of 37 and 47. The model annotated teeth 38 and 48, which is not existed (false positive). Figure 6B shows a full lower arch with a partially erupted wisdom tooth of 38. The model failed to annotate 38 (false negative). Figure 6C shows a full lower arch with two bridges (33-37 and 31-46), irregular restoration shape, and a prepared tooth of 32. The model incorrectly labelled 42 to 46 as 43 to 47 (wrong labels).

For partial-arch IOSs, Figure 6D shows a partial upper arch with a missing tooth of 14. The model annotated 14, which is not existed, and overly-annotated the gingiva at the palatal side around 15 (false-positive). Figure 6E shows a partial lower arch with three missing teeth of 41, 46, and 47. The model failed to detect tooth 35 at the scan margin (false negative). Figure 6F shows a partial lower arch with two prepared premolars of 44 and 45. The model incorrectly labelled 32 to 35 as 41, 31, 33, 34 (wrong labels).

### Correlation between dental conditions and model performance

**Fig. 7:**
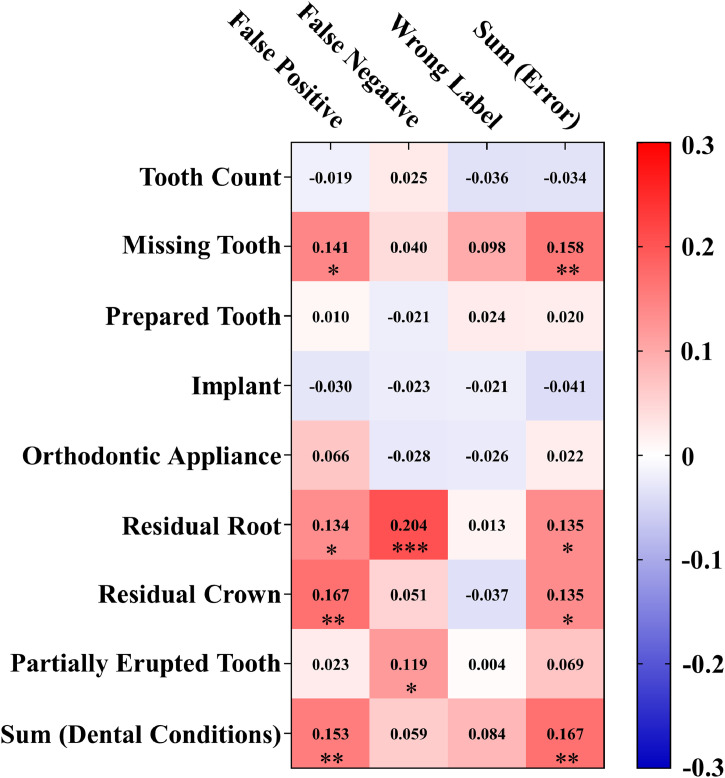
Correlation between dental conditions and prediction errors for Model 5 on partial-arch IOSs. Heatmap of Kendall’s tau-b correlation coefficients (*τ*) between dental variables and prediction errors (Model 5). Dental variables include tooth count, missing tooth, prepared tooth, implant, orthodontic appliance, residual root, residual crown, partially erupted tooth, and the total number of conditions per scan. Prediction errors include false positive, false negative, wrong label, and total errors per scan. Each cell shows the corresponding *τ* value. Asterisks indicate statistical significance: *P < .05; **P < .01; ***P < .001.

Figure 7 shows the heatmap of Kendall’s tau-b correlation coefficient (*τ*) between various dental conditions and prediction error types for Model 5 on partial-arch IOSs. Table 4 showed the *P* values of the correlation test. Correlation coefficient *τ* ranged from –0.041 to 0.204, indicating negligible to moderate correlations. The highest correlation was observed between residual roots and false negatives (*τ* = 0.204), indicating a moderate and statistically significant positive correlation (*P* = .0004, *P* < .001). Other condition–error pairs with weak and statistically significant positive correlations (*τ* = 0.10-0.20, *P* = .0014-0.0396, *P* < .05) included missing tooth–false positive, missing tooth–sum (error), residual root–false positive, residual root–sum (error), residual crown–false positive, residual crown–sum (error), partially erupted tooth–false negative, sum (dental conditions)–false positive, and sum (dental conditions)–sum (error). All remaining pairs showed |*τ*| values below 0.10 and *P* values higher than .05, suggesting a negligible and nonsignificant correlation between those dental conditions and the corresponding error types. Details of each dental conditions and prediction errors counts per scan are provided in Supplementary Material.

**Table 4:** *P* values of Kendall’s tau-b correlation test between dental variables and prediction errors (Model 5, partial-arch intraoral scans).

	FP	FN	Wrong Label	Total Errors
**Tooth Count**	0.7130	0.6281	0.4775	0.4967
**Missing Tooth**	0.0117*	0.4767	0.0772	0.0038^⁎⁎^
**Prepared Tooth**	0.8507	0.7120	0.6702	0.7120
**Implant**	0.6069	0.6903	0.7128	0.4638
**Orthodontic Appliance**	0.2484	0.6250	0.6516	0.6907
**Residual Root**	0.0197*	0.0004^⁎⁎⁎^	0.819	0.0159
**Residual Crown**	0.0036^⁎⁎^	0.3794	0.5209	0.0160*
**Partially Erupted Tooth**	0.6935	0.0396*	0.9404	0.2204
**Total Number of Conditions**	0.0045^⁎⁎^	0.2722	0.1166	0.0014^⁎⁎^

Dental variables include tooth count, missing tooth, prepared tooth, implant, orthodontic appliance, residual root, residual crown, partially erupted tooth, and the total number of conditions per scan. Prediction errors include false positive, false negative, wrong label, and total errors per scan.

Asterisks indicate statistical significance: *P < .05; **P < .01; ***P < .001.

FN, false negative; FP, false positive.

## Discussion

Tooth segmentation and labeling is a fundamental component of AI-assisted digital dentistry, serving as the foundation for downstream applications such as automated diagnosis, treatment planning, and prognosis prediction. Numerous DL-based algorithms have been developed for IOSs, with many achieving high accuracy.^29^^,^^36^ However, nearly all existing AI research has focused exclusively on full-arch IOSs. The domain of partial-arch IOSs remains largely underexplored – even for basic tasks such as tooth segmentation and labeling. For procedures such as the fabrication of computer-aided design and computer-aided manufactured crowns on prepared teeth or implants, partial-arch IOSs are widely applied as they provide higher scanning accuracy and require less operating time.^12^^,^^13^

Compared to full-arch scans, partial-arch IOSs are shorter, may lack the characteristic curvature, and often contain unclear scan boundaries or abrupt cutoffs. These limitations reduce the amount of contextual information available. Jana et al^21^ tested the performance of 10 commonly used segmentation models by training them on full-arch IOSs and evaluating them on artificially cropped partial-arch IOSs. The results showed a marked decline in segmentation performance on partial-arch data. For example, the highest tooth Dice for the front segment (0.5921), four-teeth segment (0.5722), and half-jaw segment (0.7701) were substantially lower than the highest score on full-arch scans (0.9105). These findings underscore the limitations of current models and the need for techniques adaptable to both full- and partial-arch IOSs.

In this study, ToothInstanceNet was adopted as the default model (Model 1). This two-stage model integrated low-resolution instance segmentation and label prediction with high-resolution refinement of individual tooth segmentations.^25^ ToothInstanceNet demonstrated strong performance on full-arch IOSs, achieving the highest F1-score among the five models (0.9935), with both tooth Dice and tooth macro-F1 exceeding 0.98. However, its performance on partial-arch IOSs decreased across all metrics, particularly in tooth labeling and overall effectiveness. The model showed a tooth macro-F1 of 0.7823 and a macro-IoU of 0.6537, with 639 total wrong labels, and 256 cases with error, indicating that nearly every predicted partial-arch IOS contained some labeling errors.

To address the limitations of TootInstanceNet on partial-arch IOSs, we focused on improving both the training dataset and the model pipeline. Firstly, Model 2 introduced artificially cropped partial-arch IOSs as a data augmentation strategy. The model was exposed to a broader variety of incomplete input patterns during training, simulating the clinical challenges of real partial-arch scenarios, such as incomplete curvature, fewer teeth, and limited anatomical context. As a result, improvements could be observed across all metrics on the partial-arch IOSs, most notably in tooth macro-F1 and the number of wrong labels, marking the largest improvements across the ablation study. This indicates that the model became much more capable of accurately labeling individual teeth under spatially constrained conditions, suggesting that similar improvements could be achieved in other studies even without access to real partial-arch IOSs. However, Model 2 showed a slight drop in full-arch IOSs compared to the baseline Model 1 across all key metrics, including F1-score, tooth Dice, tooth macro-F1, and macro-IoU. A likely explanation is that the introduction of cropped scans shifted the model’s focus towards learning localized features. Consequently, its capacity to make use of long-range spatial relationships was slightly reduced.

Model 3 further introduced a prealignment module to standardize IOS orientation regardless of arch completeness. The study by Alsheghri et al^27^ employed a transformer-based semantic segmentation approach for artificial partial-arch IOSs. Although their method incorporated an alignment component, it relied on a semiautomated heuristic pipeline to approximate the scan orientation. This method required additional inputs, including tooth label range or die mesh to achieve accurate registration, depending heavily on user-provided information. Zhuang et al^29^ proposed a DL-based alignment method that predicts canonical up and forward directions to normalize scan orientation. While effective for full-arch IOSs, their method assumes global anatomical completeness and does not address the challenge of left–right disambiguation, which is critical for aligning partial-arch scans. In our study, we developed a fully automated DL-based alignment module also suitable for partial-arch IOSs. Our method predicts the up and forward directions, as well as a translation vector, enabling the system to correctly reposition and orient partial scans within the dental arch. This approach allows for accurate alignment in clinically common cases where the partial-arch scan is limited to a single quadrant and lacks midline reference structures. As a result, Model 3 showed further improvements in segmentation and labeling for both full- and partial-arch IOS.

While introducing artificial partial-arch IOSs and the alignment module significantly improved the model’s performance on partial-arch IOSs, some errors persisted, particularly duplicate or skipped FDI labels. These were addressed in Model 4 by incorporating an FDI-aware postprocessing module, which leveraged both class probabilities and spatial relationships between teeth to enforce anatomical consistency. As shown in our ablation study, the transition from Model 3 (softmax-based labeling) to Model 4 (FDI-aware postprocessing) led to clear improvements in macro-IoU and tooth labeling accuracy. Since all other components remained the same, this performance gain can be attributed directly to the FDI-aware strategy, confirming its value for handling incomplete and asymmetric partial-arch IOSs.

Model 5 further included real partial-arch IOSs in the training data, which often contained irregular boundaries and diverse dental conditions, to improve the model’s generalizability and robustness in real-world applications. As a result, it achieved steady improvements across all metrics for both full-arch and partial-arch IOSs. Notably, Model 5 demonstrated excellent overall performance on partial-arch IOSs, only slightly lower than its performance on full-arch scans, with a macro-IoU of 0.9280 versus 0.9403, and number of wrong cases of 64 versus 57, respectively.

Compared to the semiautomated transformer-based approach by Alsheghri et al,^27^ which was applied to artificially cropped partial-arch IOSs and achieved tooth Dice scores between 0.936 and 0.948, our fully automated Model 5 yielded a higher tooth Dice of 0.9862. This demonstrates the effectiveness of our integrated pipeline.

Additionally, our proposed model structure also showed robust performance on full-arch IOSs using the public Teeth3DS dataset, achieving a precision, sensitivity, and F1-score above 0.99. The Teeth3DS dataset is the first large public IOS dataset comprising 1800 full-arch IOSs (upper and lower arches) from 900 patients. It provided a standardized split of 1200 scans for training and 600 scans for testing, ensuring a balanced distribution of tooth labels across scans. The Teeth3DS dataset enabled fair and reproducible comparisons across studies, serving as the evaluation reference for the MICCAI 3DTeethSeg challenge.^35^ In the 3DTeethSeg challenge, our model outperformed previous state-of-the-art methods reported by Rekik et al^36^ with the public dataset, achieving the highest values in TLA, TSA, and TIR. These results highlighted the strong generalizability of our model across both partial- and full-arch IOS cases.

Moreover, the correlation between various dental conditions and the model’s accuracy was also analysed. As Model 5 showed nearly perfect performance on full-arch IOSs (F1-score: 0.9908; tooth Dice: 0.9819; tooth macro-F1: 0.9940), correlation analysis between dental conditions and model’s error types was only performed on partial-arch IOSs. These dental conditions were considered as potential influencing factors, and they were analysed concerning different types of errors, including false positives, false negatives, wrong labels, and the total error count. As the variables did not follow a normal distribution and involved tied data, the nonparametric correlation approach, Kendall’s tau-b coefficient analysis, was adopted.

It could be found that while most correlations were weak or negligible, certain conditions, particularly residual root, residual crown, missing tooth, and partially erupted tooth, were consistently associated with increased error rates. These conditions often disrupt typical anatomical structure, introducing irregular or incomplete tooth geometries that challenge the model’s ability to segment and label. For example, residual roots may appear as small, fragmented surfaces, leading to higher rates of false negatives, and missing teeth change the expected spatial arrangement, leading to higher rates of false positives. Notably, the total number of dental conditions within a scan also showed a cumulative impact on model performance, suggesting that overall case complexity introduces significant prediction challenges.

While the Kendall’s *τ* values observed were statistically significant, their effect sizes were relatively small. Therefore, these associations should be interpreted as trends rather than strong predictors. Nonetheless, the findings provide valuable insight for future model development by identifying clinical conditions in which AI performance may be more vulnerable, where additional model refinement may be required. They could support the development of condition-aware error mitigation strategies. From a clinical perspective, partial-arch IOSs with these known challenging conditions could be flagged for manual review, thereby enhancing reliability without significantly impacting efficiency.

Failure cases further showed the model’s limitations when interpreting IOSs with complex dental conditions, including partially erupted teeth, prepared teeth, long bridge restorations, missing teeth, and irregular morphologies. Although various dental conditions were present during training, they remain difficult for the model to accurately segment and label – consistent with the correlation patterns we found, that increased case complexity might weaken the model’s accuracy.

However, this study still has several limitations. First, the real partial-arch IOSs used for training were collected from a single institution using a limited number of IOS devices. Given that IOS devices can vary in hardware specifications, processing software, and output formats, such differences may influence the model’s generalizability to other clinical environments or scanning workflows. Second, although annotations were performed twice by a single experienced dentist and verified by a second researcher, no formal intraobserver agreement was calculated. Third, no hold-out or external test dataset was used to validate the model independently. Fourth, this study focused exclusively on the permanent dentition, excluding primary and mixed dentitions to avoid ambiguity in labeling. Additionally, the model still encounters difficulties when processing IOSs with complex dental conditions, such as missing teeth or residual roots.

To address these limitations, future studies should incorporate multicentre datasets acquired with different IOS systems and scanning protocols to increase population diversity and improve model robustness across varying clinical contexts. To enhance and assess the reliability of ground truth, annotation should be conducted by multiple annotators independently, and assessments of intra- and interobserver agreement should be performed. External validation using independent datasets and further testing on real-world, low-quality scans is necessary to fully assess generalizability. Moreover, expanding the training dataset to include a broader range of dentition types and more clinically challenging cases may further improve the model’s performance in real-world clinical applications. Lastly, the further integration of condition-aware modelling techniques could enable future extensions towards multidiagnostic clinical decision-making systems.

Overall, this fully automated model offers benefits for clinical applications in general practice. At the initial stage of patient intake, the system can rapidly and accurately identify and label individual teeth from IOSs, eliminating the need for manual annotation by clinicians or dental assistants. In the future, the model could be integrated into clinical information systems, such as automated chart filing or cloud-based digital dentistry platforms. This could significantly reduce the time and effort required for clinical documentation, enabling more efficient workflows, particularly in high-volume dental practices.

Furthermore, when incorporated into comprehensive 3D dental workflows, the model has the potential to accelerate various tasks, including implant planning, computer-aided design, and computer-aided manufactured restoration design, orthodontic treatment planning, and large-scale data analysis. By supporting both full-arch and partial-arch IOSs, the model addresses a broad range of real-world clinical scenarios. Lastly, this work lays the foundation for future downstream AI applications in diagnosis, treatment planning, and longitudinal patient monitoring.

## Conclusions

The current study developed a fully automated DL model for tooth segmentation and labeling on IOSs. By incorporating artificial and real partial-arch IOSs, a DL-based alignment method, and an FDI postprocessing technique, the proposed method achieved highly accurate performance on both partial- and full-arch IOSs. By taking a significant step towards resolving current limitations in partial-arch scan analysis, the proposed approach offers a scalable solution that can be seamlessly integrated into digital dental workflows, laying the foundation for future developments in DL-driven automated dental diagnostics and treatment planning.

## Conflict of interest

The authors declare the following financial interests/personal relationships which may be considered as potential competing interests: The authors declared no potential conflicts of interest with respect to the research, authorship, and/or publication of this article. Niels van Nistelrooij and Shankeeth Vinayahalingam are employees and cofounders, respectively, of Ardim B.V., a company developing AI-assisted ultrasound technology for hip dysplasia. Ardim B.V. had no role in the design of the study; collection, analysis, and interpretation of data; or writing of the manuscript.

## Appendix Supplementary materials

Image, application 1

## Footnotes

Supplementary material associated with this article can be found in the online version at doi:10.1016/j.identj.2025.100950.
