# Supplementary material for: Fully Automated Tooth Segmentation and Labeling for Both Full- and Partial-Arch Intraoral Scans Using Deep Learning
Source: Int Dent J. 2025 Aug 14;75(5):100950. doi: 10.1016/j.identj.2025.100950 (PMC12392768; doi:10.1016/j.identj.2025.100950)
Supplement: Supplementary file 1 [file mmc1.docx]

**Supplementary Table 1.** Details of each partial-arch intraoral scan (IOS) and corresponding prediction errors (Model 5), including tooth count, dental conditions (missing tooth, prepared tooth, implant, orthodontic appliance, residual root, residual crown, and partially erupted tooth), dental conditions in total, error types (false positive, false negative, and wrong label) and errors in total. All values represent the number of teeth exhibiting the respective condition or error per scan.

| **No.** | **Tooth Count** | **Dental Conditions** | | | | | | | **Total Number of Conditions** | **Error Types** | | | **Total Errors** |
| --- | --- | --- | --- | --- | --- | --- | --- | --- | --- | --- | --- | --- | --- |
|  |  | **Missing Tooth** | **Prepared Tooth** | **Implant** | **Orthodontic Appliance** | **Residual Root** | **Residual Crown** | **Partially Erupted Tooth** |  | **False Positive** | **False Negative** | **Wrong Label** |  |
| 001 | 11 | 1 | 0 | 0 | 0 | 0 | 0 | 0 | 1 | 0 | 0 | 0 | 0 |
| 002 | 11 | 1 | 0 | 0 | 0 | 0 | 0 | 0 | 1 | 0 | 0 | 0 | 0 |
| 003 | 8 | 0 | 0 | 0 | 0 | 0 | 0 | 0 | 0 | 0 | 0 | 0 | 0 |
| 004 | 9 | 1 | 0 | 0 | 0 | 1 | 0 | 0 | 2 | 0 | 0 | 0 | 0 |
| 005 | 9 | 0 | 1 | 0 | 0 | 0 | 0 | 0 | 1 | 0 | 0 | 0 | 0 |
| 006 | 7 | 0 | 0 | 0 | 0 | 0 | 0 | 0 | 0 | 0 | 0 | 0 | 0 |
| 007 | 9 | 0 | 0 | 0 | 0 | 0 | 0 | 0 | 0 | 0 | 0 | 0 | 0 |
| 008 | 8 | 0 | 1 | 0 | 0 | 0 | 0 | 0 | 1 | 0 | 0 | 0 | 0 |
| 009 | 8 | 0 | 0 | 0 | 0 | 0 | 0 | 0 | 0 | 0 | 0 | 0 | 0 |
| 010 | 9 | 0 | 0 | 0 | 0 | 1 | 1 | 0 | 2 | 1 | 0 | 0 | 1 |
| 011 | 8 | 0 | 0 | 0 | 0 | 0 | 0 | 0 | 0 | 1 | 1 | 0 | 2 |
| 012 | 9 | 0 | 2 | 0 | 0 | 0 | 0 | 0 | 2 | 0 | 0 | 0 | 0 |
| 013 | 10 | 0 | 0 | 2 | 0 | 0 | 0 | 0 | 2 | 0 | 0 | 0 | 0 |
| 014 | 11 | 0 | 1 | 0 | 0 | 0 | 0 | 0 | 1 | 0 | 0 | 0 | 0 |
| 015 | 10 | 0 | 0 | 0 | 0 | 0 | 0 | 0 | 0 | 0 | 0 | 0 | 0 |
| 016 | 11 | 0 | 2 | 0 | 0 | 0 | 0 | 0 | 2 | 0 | 0 | 0 | 0 |
| 017 | 10 | 0 | 0 | 0 | 0 | 0 | 0 | 0 | 0 | 0 | 0 | 0 | 0 |
| 018 | 7 | 0 | 1 | 0 | 0 | 0 | 0 | 0 | 1 | 0 | 0 | 0 | 0 |
| 019 | 8 | 0 | 1 | 0 | 0 | 0 | 0 | 0 | 1 | 0 | 0 | 0 | 0 |
| 020 | 8 | 0 | 0 | 0 | 0 | 0 | 0 | 1 | 1 | 0 | 0 | 0 | 0 |
| 021 | 9 | 0 | 1 | 0 | 0 | 0 | 0 | 0 | 1 | 0 | 0 | 0 | 0 |
| 022 | 7 | 0 | 0 | 0 | 0 | 0 | 0 | 0 | 0 | 0 | 0 | 0 | 0 |
| 023 | 8 | 0 | 0 | 0 | 0 | 0 | 0 | 0 | 0 | 0 | 0 | 0 | 0 |
| 024 | 9 | 0 | 2 | 0 | 0 | 0 | 0 | 1 | 3 | 0 | 1 | 0 | 1 |
| 025 | 9 | 0 | 2 | 0 | 0 | 0 | 0 | 0 | 2 | 0 | 0 | 0 | 0 |
| 026 | 6 | 1 | 0 | 0 | 0 | 0 | 0 | 0 | 1 | 0 | 0 | 0 | 0 |
| 027 | 12 | 0 | 1 | 0 | 0 | 2 | 0 | 0 | 3 | 0 | 1 | 0 | 1 |
| 028 | 9 | 2 | 0 | 0 | 0 | 1 | 0 | 0 | 3 | 0 | 0 | 0 | 0 |
| 029 | 9 | 1 | 0 | 0 | 0 | 0 | 0 | 0 | 1 | 0 | 0 | 0 | 0 |
| 030 | 6 | 1 | 2 | 0 | 0 | 0 | 0 | 0 | 3 | 0 | 0 | 0 | 0 |
| 031 | 7 | 0 | 0 | 0 | 0 | 0 | 0 | 0 | 0 | 0 | 0 | 0 | 0 |
| 032 | 8 | 1 | 2 | 0 | 0 | 0 | 0 | 0 | 3 | 0 | 0 | 0 | 0 |
| 033 | 9 | 0 | 1 | 0 | 0 | 0 | 0 | 0 | 1 | 0 | 0 | 0 | 0 |
| 034 | 8 | 0 | 1 | 0 | 0 | 0 | 0 | 0 | 1 | 0 | 0 | 0 | 0 |
| 035 | 8 | 1 | 0 | 0 | 0 | 0 | 0 | 0 | 1 | 0 | 1 | 0 | 1 |
| 036 | 9 | 0 | 0 | 0 | 0 | 0 | 0 | 0 | 0 | 0 | 0 | 0 | 0 |
| 037 | 7 | 0 | 1 | 0 | 0 | 0 | 0 | 0 | 1 | 2 | 0 | 0 | 2 |
| 038 | 7 | 0 | 0 | 0 | 0 | 0 | 0 | 0 | 0 | 0 | 0 | 0 | 0 |
| 039 | 10 | 1 | 0 | 0 | 0 | 0 | 0 | 0 | 1 | 0 | 0 | 0 | 0 |
| 040 | 9 | 0 | 0 | 0 | 0 | 0 | 0 | 0 | 0 | 0 | 0 | 0 | 0 |
| 041 | 8 | 0 | 1 | 0 | 0 | 0 | 0 | 0 | 1 | 0 | 0 | 0 | 0 |
| 042 | 9 | 0 | 1 | 0 | 0 | 0 | 0 | 0 | 1 | 0 | 0 | 0 | 0 |
| 043 | 10 | 0 | 0 | 0 | 0 | 0 | 0 | 0 | 0 | 0 | 0 | 0 | 0 |
| 044 | 8 | 2 | 0 | 0 | 0 | 0 | 0 | 0 | 2 | 0 | 0 | 0 | 0 |
| 045 | 9 | 0 | 0 | 0 | 0 | 0 | 0 | 0 | 0 | 0 | 0 | 0 | 0 |
| 046 | 8 | 0 | 1 | 0 | 0 | 0 | 0 | 0 | 1 | 0 | 0 | 0 | 0 |
| 047 | 11 | 0 | 0 | 0 | 0 | 0 | 0 | 0 | 0 | 1 | 0 | 0 | 1 |
| 048 | 8 | 1 | 0 | 0 | 0 | 0 | 0 | 0 | 1 | 1 | 0 | 0 | 1 |
| 049 | 10 | 0 | 1 | 0 | 0 | 0 | 0 | 0 | 1 | 0 | 0 | 0 | 0 |
| 050 | 8 | 0 | 0 | 0 | 0 | 0 | 0 | 0 | 0 | 0 | 0 | 0 | 0 |
| 051 | 10 | 0 | 0 | 0 | 0 | 0 | 0 | 0 | 0 | 1 | 0 | 0 | 1 |
| 052 | 10 | 0 | 0 | 0 | 0 | 0 | 0 | 0 | 0 | 0 | 0 | 0 | 0 |
| 053 | 9 | 0 | 1 | 0 | 0 | 0 | 0 | 0 | 1 | 0 | 0 | 0 | 0 |
| 054 | 7 | 0 | 0 | 0 | 0 | 0 | 0 | 0 | 0 | 0 | 0 | 0 | 0 |
| 055 | 6 | 0 | 0 | 0 | 0 | 0 | 0 | 0 | 0 | 0 | 0 | 0 | 0 |
| 056 | 7 | 0 | 1 | 0 | 0 | 0 | 0 | 0 | 1 | 0 | 0 | 0 | 0 |
| 057 | 7 | 0 | 0 | 0 | 0 | 0 | 0 | 0 | 0 | 0 | 1 | 0 | 1 |
| 058 | 6 | 0 | 0 | 0 | 0 | 0 | 0 | 0 | 0 | 0 | 0 | 0 | 0 |
| 059 | 5 | 0 | 1 | 0 | 0 | 0 | 0 | 0 | 1 | 1 | 0 | 3 | 4 |
| 060 | 5 | 1 | 0 | 0 | 0 | 0 | 0 | 0 | 1 | 0 | 0 | 1 | 1 |
| 061 | 10 | 0 | 0 | 0 | 0 | 0 | 0 | 0 | 0 | 0 | 0 | 0 | 0 |
| 062 | 8 | 0 | 1 | 0 | 0 | 0 | 0 | 0 | 1 | 0 | 0 | 0 | 0 |
| 063 | 7 | 1 | 1 | 0 | 0 | 0 | 0 | 1 | 3 | 0 | 0 | 1 | 1 |
| 064 | 8 | 1 | 0 | 0 | 0 | 0 | 0 | 1 | 2 | 0 | 0 | 0 | 0 |
| 065 | 6 | 2 | 0 | 0 | 0 | 0 | 0 | 0 | 2 | 0 | 0 | 0 | 0 |
| 066 | 8 | 0 | 0 | 0 | 0 | 0 | 0 | 0 | 0 | 0 | 0 | 0 | 0 |
| 067 | 10 | 1 | 1 | 0 | 0 | 0 | 0 | 0 | 2 | 0 | 0 | 0 | 0 |
| 068 | 9 | 0 | 0 | 0 | 0 | 0 | 0 | 0 | 0 | 0 | 0 | 0 | 0 |
| 069 | 4 | 1 | 0 | 0 | 0 | 0 | 0 | 0 | 1 | 1 | 0 | 4 | 5 |
| 070 | 6 | 0 | 0 | 0 | 0 | 0 | 0 | 0 | 0 | 0 | 0 | 6 | 6 |
| 071 | 6 | 2 | 1 | 0 | 0 | 0 | 0 | 0 | 3 | 0 | 0 | 0 | 0 |
| 072 | 6 | 0 | 0 | 0 | 0 | 0 | 0 | 0 | 0 | 0 | 0 | 0 | 0 |
| 073 | 6 | 0 | 0 | 0 | 0 | 0 | 0 | 0 | 0 | 0 | 0 | 0 | 0 |
| 074 | 8 | 0 | 1 | 0 | 0 | 0 | 0 | 1 | 2 | 0 | 0 | 0 | 0 |
| 075 | 8 | 1 | 0 | 0 | 0 | 0 | 0 | 0 | 1 | 0 | 0 | 0 | 0 |
| 076 | 9 | 0 | 2 | 0 | 0 | 0 | 0 | 0 | 2 | 0 | 0 | 0 | 0 |
| 077 | 8 | 0 | 0 | 0 | 0 | 0 | 1 | 0 | 1 | 1 | 0 | 0 | 1 |
| 078 | 8 | 0 | 1 | 0 | 0 | 0 | 0 | 0 | 1 | 0 | 0 | 0 | 0 |
| 079 | 8 | 0 | 1 | 0 | 0 | 0 | 0 | 0 | 1 | 0 | 0 | 0 | 0 |
| 080 | 8 | 0 | 0 | 0 | 0 | 0 | 0 | 0 | 0 | 0 | 0 | 0 | 0 |
| 081 | 8 | 1 | 0 | 0 | 0 | 0 | 0 | 0 | 1 | 0 | 0 | 2 | 2 |
| 082 | 8 | 0 | 1 | 0 | 0 | 0 | 0 | 0 | 1 | 0 | 0 | 0 | 0 |
| 083 | 6 | 0 | 1 | 0 | 0 | 0 | 0 | 0 | 1 | 0 | 0 | 0 | 0 |
| 084 | 6 | 0 | 0 | 0 | 0 | 0 | 0 | 0 | 0 | 0 | 0 | 0 | 0 |
| 085 | 6 | 0 | 0 | 0 | 0 | 0 | 0 | 0 | 0 | 0 | 0 | 0 | 0 |
| 086 | 7 | 0 | 1 | 0 | 0 | 0 | 0 | 0 | 1 | 0 | 0 | 0 | 0 |
| 087 | 7 | 0 | 2 | 0 | 0 | 0 | 0 | 0 | 2 | 0 | 0 | 4 | 4 |
| 088 | 7 | 1 | 0 | 0 | 0 | 0 | 0 | 0 | 1 | 1 | 0 | 0 | 1 |
| 089 | 8 | 1 | 0 | 0 | 0 | 0 | 0 | 0 | 1 | 0 | 0 | 2 | 2 |
| 090 | 8 | 1 | 1 | 0 | 0 | 0 | 0 | 0 | 2 | 0 | 0 | 0 | 0 |
| 091 | 7 | 0 | 1 | 0 | 0 | 0 | 0 | 0 | 1 | 1 | 0 | 0 | 1 |
| 092 | 8 | 0 | 0 | 0 | 0 | 0 | 0 | 0 | 0 | 0 | 0 | 0 | 0 |
| 093 | 7 | 1 | 0 | 0 | 0 | 0 | 0 | 0 | 1 | 0 | 0 | 0 | 0 |
| 094 | 8 | 0 | 1 | 0 | 0 | 0 | 0 | 0 | 1 | 0 | 0 | 0 | 0 |
| 095 | 7 | 0 | 0 | 0 | 0 | 0 | 0 | 0 | 0 | 0 | 0 | 0 | 0 |
| 096 | 7 | 0 | 1 | 0 | 0 | 0 | 0 | 0 | 1 | 0 | 0 | 0 | 0 |
| 097 | 9 | 0 | 0 | 0 | 0 | 0 | 0 | 0 | 0 | 0 | 0 | 0 | 0 |
| 098 | 8 | 0 | 2 | 0 | 0 | 0 | 0 | 0 | 2 | 0 | 0 | 0 | 0 |
| 099 | 14 | 0 | 0 | 0 | 0 | 0 | 0 | 0 | 0 | 0 | 0 | 0 | 0 |
| 100 | 11 | 0 | 4 | 0 | 0 | 0 | 0 | 0 | 4 | 0 | 0 | 0 | 0 |
| 101 | 12 | 0 | 0 | 0 | 0 | 0 | 0 | 0 | 0 | 0 | 0 | 0 | 0 |
| 102 | 9 | 1 | 4 | 0 | 0 | 0 | 0 | 0 | 5 | 0 | 0 | 0 | 0 |
| 103 | 8 | 0 | 0 | 0 | 0 | 0 | 0 | 0 | 0 | 0 | 0 | 0 | 0 |
| 104 | 7 | 0 | 1 | 0 | 0 | 0 | 0 | 0 | 1 | 0 | 0 | 0 | 0 |
| 105 | 11 | 0 | 1 | 0 | 0 | 0 | 0 | 0 | 1 | 0 | 0 | 0 | 0 |
| 106 | 8 | 0 | 0 | 0 | 0 | 0 | 0 | 0 | 0 | 0 | 0 | 0 | 0 |
| 107 | 9 | 1 | 0 | 0 | 0 | 0 | 0 | 0 | 1 | 0 | 0 | 0 | 0 |
| 108 | 8 | 0 | 0 | 0 | 0 | 0 | 0 | 0 | 0 | 0 | 0 | 0 | 0 |
| 109 | 9 | 0 | 1 | 0 | 0 | 0 | 0 | 1 | 2 | 0 | 0 | 0 | 0 |
| 110 | 8 | 0 | 0 | 0 | 0 | 0 | 0 | 0 | 0 | 0 | 0 | 0 | 0 |
| 111 | 8 | 0 | 1 | 0 | 0 | 0 | 0 | 0 | 1 | 0 | 0 | 0 | 0 |
| 112 | 9 | 0 | 0 | 0 | 0 | 0 | 0 | 0 | 0 | 0 | 0 | 0 | 0 |
| 113 | 9 | 0 | 0 | 0 | 0 | 0 | 0 | 0 | 0 | 1 | 0 | 0 | 1 |
| 114 | 8 | 0 | 1 | 0 | 0 | 0 | 0 | 0 | 1 | 0 | 0 | 0 | 0 |
| 115 | 9 | 0 | 1 | 0 | 0 | 0 | 0 | 0 | 1 | 0 | 0 | 0 | 0 |
| 116 | 8 | 0 | 0 | 0 | 0 | 0 | 0 | 0 | 0 | 0 | 0 | 0 | 0 |
| 117 | 8 | 0 | 0 | 0 | 0 | 0 | 0 | 0 | 0 | 0 | 0 | 0 | 0 |
| 118 | 8 | 0 | 2 | 0 | 0 | 0 | 0 | 0 | 2 | 0 | 0 | 0 | 0 |
| 119 | 7 | 0 | 0 | 0 | 0 | 0 | 0 | 0 | 0 | 0 | 0 | 0 | 0 |
| 120 | 7 | 0 | 1 | 0 | 0 | 0 | 0 | 0 | 1 | 0 | 0 | 0 | 0 |
| 121 | 10 | 0 | 0 | 0 | 0 | 0 | 0 | 0 | 0 | 0 | 0 | 0 | 0 |
| 122 | 10 | 0 | 1 | 0 | 0 | 0 | 0 | 0 | 1 | 0 | 0 | 0 | 0 |
| 123 | 8 | 2 | 0 | 0 | 0 | 0 | 0 | 0 | 2 | 0 | 0 | 0 | 0 |
| 124 | 8 | 0 | 0 | 0 | 0 | 0 | 0 | 0 | 0 | 0 | 0 | 0 | 0 |
| 125 | 11 | 1 | 0 | 0 | 0 | 0 | 0 | 0 | 1 | 0 | 0 | 0 | 0 |
| 126 | 10 | 1 | 0 | 0 | 0 | 0 | 0 | 0 | 1 | 0 | 0 | 0 | 0 |
| 127 | 7 | 0 | 0 | 0 | 0 | 0 | 0 | 0 | 0 | 0 | 0 | 0 | 0 |
| 128 | 8 | 0 | 2 | 0 | 0 | 0 | 0 | 0 | 2 | 0 | 0 | 0 | 0 |
| 129 | 7 | 0 | 1 | 0 | 0 | 0 | 0 | 0 | 1 | 0 | 0 | 0 | 0 |
| 130 | 8 | 0 | 0 | 0 | 0 | 0 | 0 | 0 | 0 | 0 | 0 | 0 | 0 |
| 131 | 8 | 0 | 1 | 0 | 0 | 0 | 0 | 0 | 1 | 0 | 0 | 0 | 0 |
| 132 | 7 | 0 | 0 | 0 | 0 | 0 | 0 | 0 | 0 | 0 | 0 | 0 | 0 |
| 133 | 12 | 0 | 0 | 0 | 0 | 0 | 0 | 0 | 0 | 0 | 0 | 0 | 0 |
| 134 | 12 | 0 | 1 | 0 | 0 | 0 | 0 | 0 | 1 | 0 | 0 | 0 | 0 |
| 135 | 8 | 0 | 1 | 0 | 0 | 0 | 0 | 0 | 1 | 0 | 0 | 2 | 2 |
| 136 | 8 | 0 | 0 | 0 | 0 | 0 | 0 | 0 | 0 | 0 | 0 | 0 | 0 |
| 137 | 10 | 0 | 0 | 0 | 0 | 0 | 0 | 0 | 0 | 0 | 0 | 0 | 0 |
| 138 | 7 | 0 | 1 | 0 | 0 | 0 | 0 | 0 | 1 | 0 | 0 | 0 | 0 |
| 139 | 9 | 0 | 0 | 0 | 0 | 0 | 0 | 0 | 0 | 0 | 0 | 0 | 0 |
| 140 | 6 | 1 | 0 | 0 | 0 | 0 | 0 | 0 | 1 | 0 | 0 | 0 | 0 |
| 141 | 11 | 0 | 0 | 0 | 0 | 0 | 0 | 0 | 0 | 0 | 0 | 0 | 0 |
| 142 | 10 | 0 | 0 | 0 | 0 | 0 | 0 | 0 | 0 | 0 | 0 | 3 | 3 |
| 143 | 7 | 3 | 0 | 0 | 0 | 0 | 0 | 0 | 3 | 1 | 1 | 0 | 2 |
| 144 | 7 | 0 | 0 | 0 | 0 | 0 | 0 | 0 | 0 | 0 | 1 | 0 | 1 |
| 145 | 8 | 0 | 0 | 0 | 0 | 0 | 0 | 0 | 0 | 0 | 0 | 0 | 0 |
| 146 | 6 | 3 | 0 | 0 | 0 | 0 | 0 | 0 | 3 | 1 | 0 | 0 | 1 |
| 147 | 9 | 0 | 1 | 0 | 0 | 0 | 0 | 0 | 1 | 0 | 0 | 0 | 0 |
| 148 | 8 | 0 | 0 | 0 | 0 | 0 | 0 | 0 | 0 | 0 | 0 | 0 | 0 |
| 149 | 10 | 1 | 3 | 0 | 0 | 0 | 0 | 0 | 4 | 1 | 0 | 0 | 1 |
| 150 | 10 | 1 | 0 | 0 | 0 | 1 | 0 | 0 | 2 | 1 | 0 | 0 | 1 |
| 151 | 8 | 1 | 0 | 0 | 0 | 0 | 0 | 0 | 1 | 0 | 1 | 0 | 1 |
| 152 | 8 | 0 | 0 | 0 | 0 | 0 | 0 | 0 | 0 | 0 | 0 | 0 | 0 |
| 153 | 8 | 0 | 1 | 0 | 0 | 0 | 0 | 0 | 1 | 1 | 0 | 0 | 1 |
| 154 | 7 | 0 | 0 | 0 | 0 | 0 | 0 | 0 | 0 | 0 | 0 | 0 | 0 |
| 155 | 9 | 0 | 0 | 0 | 0 | 0 | 0 | 0 | 0 | 0 | 0 | 0 | 0 |
| 156 | 8 | 1 | 0 | 0 | 0 | 0 | 0 | 0 | 1 | 0 | 0 | 0 | 0 |
| 157 | 8 | 1 | 0 | 0 | 0 | 0 | 0 | 0 | 1 | 0 | 0 | 1 | 1 |
| 158 | 8 | 0 | 1 | 0 | 0 | 0 | 0 | 1 | 2 | 0 | 0 | 0 | 0 |
| 159 | 8 | 0 | 0 | 0 | 0 | 0 | 0 | 0 | 0 | 0 | 0 | 0 | 0 |
| 160 | 8 | 1 | 0 | 0 | 0 | 0 | 0 | 0 | 1 | 0 | 1 | 0 | 1 |
| 161 | 8 | 0 | 0 | 0 | 0 | 0 | 0 | 0 | 0 | 0 | 0 | 0 | 0 |
| 162 | 9 | 0 | 1 | 0 | 0 | 0 | 0 | 1 | 2 | 0 | 0 | 0 | 0 |
| 163 | 9 | 2 | 0 | 0 | 0 | 0 | 0 | 0 | 2 | 0 | 2 | 4 | 6 |
| 164 | 9 | 1 | 0 | 0 | 0 | 0 | 0 | 0 | 1 | 0 | 0 | 0 | 0 |
| 165 | 9 | 1 | 0 | 0 | 0 | 0 | 0 | 0 | 1 | 0 | 0 | 0 | 0 |
| 166 | 10 | 0 | 0 | 0 | 0 | 0 | 0 | 0 | 0 | 0 | 0 | 0 | 0 |
| 167 | 9 | 0 | 0 | 0 | 0 | 0 | 0 | 0 | 0 | 0 | 0 | 0 | 0 |
| 168 | 9 | 0 | 2 | 0 | 0 | 0 | 0 | 0 | 2 | 0 | 0 | 0 | 0 |
| 169 | 11 | 1 | 0 | 0 | 0 | 0 | 0 | 0 | 1 | 1 | 0 | 0 | 1 |
| 170 | 9 | 1 | 0 | 0 | 0 | 0 | 0 | 0 | 1 | 0 | 0 | 0 | 0 |
| 171 | 10 | 2 | 0 | 0 | 0 | 0 | 0 | 0 | 2 | 0 | 0 | 6 | 6 |
| 172 | 9 | 1 | 0 | 0 | 0 | 0 | 0 | 0 | 1 | 0 | 0 | 0 | 0 |
| 173 | 9 | 3 | 3 | 0 | 0 | 0 | 0 | 0 | 6 | 0 | 0 | 1 | 1 |
| 174 | 11 | 1 | 0 | 0 | 0 | 0 | 0 | 0 | 1 | 0 | 0 | 0 | 0 |
| 175 | 12 | 0 | 0 | 0 | 0 | 0 | 0 | 0 | 0 | 0 | 0 | 0 | 0 |
| 176 | 7 | 0 | 1 | 0 | 0 | 0 | 0 | 0 | 1 | 0 | 0 | 0 | 0 |
| 177 | 7 | 0 | 0 | 0 | 0 | 0 | 0 | 0 | 0 | 0 | 0 | 0 | 0 |
| 178 | 8 | 0 | 1 | 0 | 0 | 0 | 0 | 0 | 1 | 0 | 0 | 0 | 0 |
| 179 | 9 | 0 | 1 | 0 | 0 | 0 | 0 | 1 | 2 | 1 | 0 | 0 | 1 |
| 180 | 8 | 0 | 0 | 0 | 0 | 0 | 0 | 1 | 1 | 0 | 1 | 0 | 1 |
| 181 | 10 | 0 | 0 | 0 | 0 | 0 | 0 | 0 | 0 | 0 | 0 | 0 | 0 |
| 182 | 7 | 0 | 2 | 0 | 0 | 0 | 0 | 0 | 0 | 0 | 0 | 0 | 0 |
| 183 | 10 | 0 | 1 | 0 | 0 | 0 | 0 | 0 | 1 | 0 | 0 | 0 | 0 |
| 184 | 9 | 0 | 0 | 0 | 0 | 0 | 0 | 0 | 0 | 0 | 0 | 0 | 0 |
| 185 | 8 | 0 | 2 | 0 | 0 | 0 | 0 | 0 | 2 | 0 | 0 | 0 | 0 |
| 186 | 7 | 2 | 0 | 1 | 0 | 0 | 0 | 0 | 3 | 0 | 0 | 0 | 0 |
| 187 | 9 | 0 | 0 | 0 | 0 | 0 | 0 | 0 | 0 | 0 | 0 | 0 | 0 |
| 188 | 10 | 0 | 2 | 0 | 0 | 1 | 1 | 0 | 2 | 0 | 1 | 0 | 1 |
| 189 | 7 | 1 | 0 | 0 | 0 | 0 | 0 | 0 | 1 | 0 | 1 | 0 | 1 |
| 190 | 8 | 1 | 2 | 0 | 0 | 0 | 0 | 0 | 3 | 0 | 0 | 0 | 0 |
| 191 | 9 | 0 | 1 | 0 | 0 | 0 | 0 | 0 | 1 | 0 | 0 | 3 | 3 |
| 192 | 9 | 0 | 0 | 0 | 0 | 1 | 0 | 0 | 1 | 0 | 0 | 0 | 0 |
| 193 | 9 | 0 | 2 | 0 | 0 | 0 | 0 | 1 | 3 | 2 | 1 | 0 | 3 |
| 194 | 8 | 0 | 0 | 0 | 0 | 0 | 1 | 0 | 1 | 0 | 0 | 0 | 0 |
| 195 | 9 | 0 | 0 | 0 | 0 | 0 | 0 | 0 | 0 | 0 | 0 | 0 | 0 |
| 196 | 10 | 0 | 1 | 0 | 0 | 0 | 0 | 0 | 1 | 0 | 0 | 0 | 0 |
| 197 | 10 | 1 | 0 | 0 | 0 | 0 | 0 | 0 | 1 | 0 | 0 | 0 | 0 |
| 198 | 11 | 0 | 1 | 0 | 0 | 0 | 0 | 0 | 1 | 0 | 0 | 0 | 0 |
| 199 | 11 | 0 | 1 | 0 | 0 | 0 | 0 | 1 | 2 | 0 | 0 | 0 | 0 |
| 200 | 11 | 0 | 1 | 0 | 0 | 0 | 0 | 1 | 2 | 0 | 0 | 0 | 0 |
| 201 | 9 | 0 | 0 | 0 | 0 | 0 | 0 | 0 | 0 | 0 | 0 | 0 | 0 |
| 202 | 8 | 0 | 1 | 0 | 0 | 0 | 0 | 0 | 1 | 0 | 0 | 0 | 0 |
| 203 | 10 | 0 | 1 | 0 | 0 | 0 | 0 | 0 | 1 | 0 | 0 | 0 | 0 |
| 204 | 9 | 0 | 0 | 0 | 0 | 0 | 0 | 0 | 0 | 0 | 0 | 0 | 0 |
| 205 | 11 | 0 | 1 | 0 | 0 | 0 | 0 | 0 | 1 | 0 | 0 | 0 | 0 |
| 206 | 8 | 0 | 0 | 0 | 0 | 0 | 0 | 0 | 0 | 0 | 1 | 0 | 1 |
| 207 | 9 | 0 | 1 | 0 | 0 | 0 | 0 | 0 | 1 | 0 | 0 | 0 | 0 |
| 208 | 8 | 0 | 0 | 0 | 0 | 0 | 0 | 0 | 0 | 0 | 0 | 0 | 0 |
| 209 | 10 | 0 | 1 | 0 | 0 | 0 | 0 | 0 | 1 | 0 | 0 | 0 | 0 |
| 210 | 9 | 0 | 1 | 0 | 0 | 0 | 0 | 0 | 1 | 0 | 0 | 0 | 0 |
| 211 | 13 | 0 | 0 | 0 | 0 | 0 | 0 | 0 | 0 | 0 | 0 | 0 | 0 |
| 212 | 9 | 0 | 1 | 0 | 0 | 0 | 0 | 0 | 1 | 0 | 0 | 0 | 0 |
| 213 | 9 | 0 | 1 | 0 | 0 | 0 | 0 | 0 | 1 | 0 | 0 | 0 | 0 |
| 214 | 8 | 0 | 0 | 0 | 0 | 0 | 0 | 0 | 0 | 0 | 0 | 0 | 0 |
| 215 | 10 | 1 | 0 | 0 | 0 | 0 | 0 | 1 | 2 | 0 | 0 | 0 | 0 |
| 216 | 10 | 1 | 1 | 0 | 0 | 0 | 0 | 0 | 2 | 0 | 0 | 0 | 0 |
| 217 | 8 | 0 | 0 | 0 | 0 | 0 | 0 | 0 | 0 | 0 | 0 | 0 | 0 |
| 218 | 9 | 0 | 1 | 0 | 0 | 0 | 0 | 0 | 1 | 0 | 0 | 0 | 0 |
| 219 | 9 | 0 | 0 | 0 | 0 | 0 | 0 | 0 | 0 | 0 | 0 | 0 | 0 |
| 220 | 9 | 0 | 1 | 0 | 0 | 0 | 0 | 0 | 1 | 0 | 0 | 0 | 0 |
| 221 | 8 | 0 | 1 | 0 | 0 | 0 | 0 | 0 | 1 | 0 | 0 | 0 | 0 |
| 222 | 7 | 0 | 0 | 0 | 0 | 0 | 0 | 0 | 0 | 0 | 0 | 0 | 0 |
| 223 | 7 | 0 | 0 | 0 | 0 | 0 | 0 | 0 | 0 | 0 | 0 | 0 | 0 |
| 224 | 8 | 0 | 1 | 0 | 0 | 0 | 0 | 0 | 1 | 0 | 0 | 0 | 0 |
| 225 | 9 | 0 | 2 | 0 | 0 | 1 | 0 | 0 | 3 | 0 | 0 | 0 | 0 |
| 226 | 9 | 0 | 0 | 0 | 0 | 1 | 0 | 0 | 1 | 0 | 0 | 0 | 0 |
| 227 | 9 | 1 | 0 | 0 | 0 | 0 | 0 | 0 | 1 | 0 | 0 | 0 | 0 |
| 228 | 8 | 0 | 0 | 0 | 0 | 0 | 0 | 0 | 0 | 0 | 0 | 0 | 0 |
| 229 | 7 | 1 | 0 | 0 | 0 | 0 | 0 | 0 | 1 | 0 | 0 | 0 | 0 |
| 230 | 8 | 0 | 0 | 0 | 0 | 0 | 0 | 0 | 0 | 0 | 0 | 0 | 0 |
| 231 | 12 | 1 | 1 | 0 | 0 | 0 | 0 | 0 | 2 | 1 | 0 | 0 | 1 |
| 232 | 12 | 1 | 0 | 0 | 0 | 1 | 0 | 0 | 2 | 1 | 1 | 0 | 2 |
| 233 | 10 | 0 | 0 | 0 | 0 | 0 | 0 | 0 | 0 | 0 | 0 | 0 | 0 |
| 234 | 8 | 1 | 0 | 0 | 0 | 0 | 0 | 0 | 1 | 0 | 0 | 0 | 0 |
| 235 | 10 | 0 | 1 | 0 | 0 | 0 | 0 | 0 | 1 | 1 | 0 | 0 | 1 |
| 236 | 7 | 1 | 0 | 0 | 0 | 0 | 0 | 0 | 1 | 0 | 0 | 0 | 0 |
| 237 | 10 | 1 | 0 | 0 | 0 | 0 | 0 | 0 | 1 | 0 | 0 | 0 | 0 |
| 238 | 11 | 0 | 0 | 0 | 0 | 0 | 0 | 0 | 0 | 0 | 0 | 0 | 0 |
| 239 | 9 | 1 | 0 | 0 | 0 | 0 | 0 | 0 | 1 | 0 | 0 | 0 | 0 |
| 240 | 9 | 0 | 0 | 0 | 0 | 0 | 0 | 0 | 0 | 0 | 0 | 0 | 0 |
| 241 | 11 | 1 | 0 | 0 | 6 | 0 | 0 | 0 | 7 | 0 | 0 | 0 | 0 |
| 242 | 13 | 0 | 4 | 0 | 0 | 0 | 1 | 0 | 5 | 1 | 0 | 0 | 1 |
| 243 | 10 | 1 | 2 | 0 | 0 | 0 | 0 | 0 | 3 | 0 | 0 | 0 | 0 |
| 244 | 12 | 0 | 0 | 0 | 0 | 0 | 0 | 0 | 0 | 0 | 0 | 3 | 3 |
| 245 | 9 | 0 | 0 | 0 | 0 | 0 | 0 | 0 | 0 | 0 | 0 | 0 | 0 |
| 246 | 8 | 0 | 1 | 0 | 0 | 0 | 0 | 0 | 1 | 0 | 0 | 0 | 0 |
| 247 | 7 | 1 | 0 | 0 | 0 | 0 | 0 | 0 | 1 | 0 | 0 | 0 | 0 |
| 248 | 9 | 0 | 0 | 0 | 0 | 0 | 0 | 0 | 0 | 0 | 0 | 0 | 0 |
| 249 | 9 | 1 | 0 | 0 | 0 | 0 | 0 | 0 | 1 | 0 | 0 | 0 | 0 |
| 250 | 11 | 0 | 0 | 0 | 0 | 0 | 0 | 0 | 0 | 0 | 0 | 0 | 0 |
| 251 | 9 | 0 | 0 | 0 | 0 | 0 | 0 | 0 | 0 | 1 | 0 | 0 | 1 |
| 252 | 8 | 0 | 1 | 0 | 0 | 0 | 0 | 0 | 1 | 0 | 1 | 0 | 1 |
| 253 | 12 | 0 | 0 | 0 | 0 | 0 | 0 | 0 | 0 | 0 | 0 | 0 | 0 |
| 254 | 9 | 0 | 1 | 0 | 0 | 0 | 0 | 0 | 1 | 0 | 0 | 0 | 0 |
| 255 | 11 | 0 | 2 | 0 | 0 | 0 | 0 | 0 | 2 | 0 | 0 | 4 | 4 |
| 256 | 10 | 0 | 0 | 0 | 0 | 0 | 0 | 0 | 0 | 0 | 0 | 0 | 0 |
| 257 | 12 | 0 | 0 | 0 | 0 | 0 | 0 | 0 | 0 | 0 | 0 | 0 | 0 |
| 258 | 12 | 0 | 2 | 0 | 0 | 0 | 0 | 0 | 0 | 0 | 0 | 0 | 0 |
| 259 | 9 | 1 | 0 | 0 | 0 | 0 | 0 | 0 | 1 | 1 | 0 | 0 | 1 |
| 260 | 7 | 1 | 0 | 0 | 0 | 0 | 0 | 0 | 1 | 2 | 0 | 0 | 2 |
| 261 | 7 | 3 | 0 | 0 | 0 | 0 | 0 | 0 | 3 | 1 | 0 | 0 | 1 |
| 262 | 8 | 3 | 0 | 0 | 0 | 0 | 0 | 0 | 3 | 0 | 0 | 0 | 0 |
| 263 | 9 | 0 | 0 | 0 | 0 | 0 | 0 | 0 | 0 | 0 | 1 | 5 | 6 |
| 264 | 9 | 1 | 0 | 0 | 0 | 0 | 0 | 0 | 1 | 0 | 0 | 0 | 0 |
| 265 | 10 | 0 | 0 | 0 | 0 | 0 | 0 | 0 | 0 | 0 | 0 | 0 | 0 |
| 266 | 9 | 2 | 0 | 0 | 0 | 0 | 0 | 0 | 2 | 0 | 0 | 0 | 0 |
| 267 | 12 | 0 | 0 | 0 | 0 | 1 | 0 | 0 | 1 | 1 | 1 | 1 | 3 |
| 268 | 10 | 1 | 2 | 0 | 0 | 0 | 0 | 0 | 1 | 1 | 1 | 0 | 2 |
| 269 | 11 | 0 | 0 | 0 | 0 | 0 | 0 | 0 | 0 | 0 | 0 | 0 | 0 |
| 270 | 7 | 2 | 0 | 0 | 0 | 0 | 0 | 0 | 2 | 0 | 0 | 0 | 0 |
| 271 | 11 | 0 | 0 | 0 | 0 | 0 | 0 | 0 | 0 | 0 | 0 | 0 | 0 |
| 272 | 6 | 1 | 0 | 0 | 0 | 0 | 0 | 0 | 1 | 0 | 0 | 0 | 0 |
| 273 | 9 | 1 | 0 | 0 | 8 | 0 | 0 | 0 | 9 | 0 | 0 | 0 | 0 |
| 274 | 7 | 1 | 1 | 0 | 7 | 0 | 0 | 0 | 2 | 1 | 0 | 0 | 1 |
| 275 | 8 | 0 | 0 | 0 | 0 | 0 | 0 | 0 | 0 | 0 | 0 | 0 | 0 |
| 276 | 7 | 1 | 0 | 0 | 0 | 0 | 0 | 0 | 1 | 0 | 0 | 0 | 0 |
| 277 | 8 | 1 | 0 | 0 | 0 | 0 | 0 | 0 | 1 | 0 | 0 | 0 | 0 |
| 278 | 6 | 0 | 0 | 0 | 0 | 0 | 0 | 0 | 0 | 0 | 0 | 0 | 0 |
| 279 | 10 | 0 | 0 | 0 | 0 | 0 | 0 | 0 | 0 | 0 | 0 | 0 | 0 |
| 280 | 6 | 2 | 0 | 0 | 0 | 0 | 0 | 0 | 2 | 0 | 0 | 0 | 0 |
| 281 | 10 | 1 | 0 | 0 | 0 | 0 | 0 | 0 | 1 | 0 | 0 | 0 | 0 |
| 282 | 10 | 0 | 0 | 0 | 0 | 0 | 0 | 0 | 0 | 0 | 0 | 0 | 0 |
| 283 | 6 | 2 | 0 | 0 | 0 | 0 | 0 | 0 | 2 | 1 | 0 | 0 | 1 |
| 284 | 7 | 1 | 0 | 0 | 0 | 0 | 0 | 0 | 1 | 0 | 0 | 0 | 0 |
| 285 | 11 | 0 | 0 | 0 | 0 | 0 | 0 | 0 | 0 | 0 | 0 | 0 | 0 |
| 286 | 10 | 1 | 0 | 0 | 0 | 0 | 0 | 0 | 1 | 0 | 0 | 0 | 0 |
| 287 | 8 | 1 | 0 | 0 | 0 | 0 | 0 | 0 | 1 | 1 | 0 | 0 | 1 |
| 288 | 8 | 0 | 0 | 0 | 0 | 0 | 0 | 0 | 0 | 0 | 0 | 0 | 0 |
| 289 | 9 | 0 | 0 | 0 | 0 | 0 | 0 | 0 | 0 | 0 | 0 | 0 | 0 |
| 290 | 8 | 1 | 0 | 0 | 0 | 0 | 0 | 0 | 1 | 0 | 0 | 0 | 0 |
| 291 | 13 | 0 | 0 | 0 | 0 | 0 | 1 | 0 | 1 | 0 | 0 | 0 | 0 |
| 292 | 11 | 1 | 0 | 0 | 0 | 1 | 0 | 0 | 2 | 0 | 0 | 0 | 0 |
| 293 | 9 | 0 | 0 | 0 | 0 | 0 | 0 | 0 | 0 | 1 | 1 | 0 | 2 |
| 294 | 7 | 2 | 0 | 0 | 0 | 0 | 0 | 0 | 2 | 0 | 0 | 0 | 0 |
| 295 | 13 | 0 | 0 | 0 | 0 | 0 | 0 | 0 | 0 | 0 | 1 | 0 | 1 |
| 296 | 10 | 2 | 0 | 0 | 0 | 0 | 0 | 0 | 0 | 0 | 0 | 0 | 0 |
| 297 | 11 | 1 | 0 | 0 | 0 | 0 | 0 | 0 | 1 | 0 | 0 | 0 | 0 |
| 298 | 11 | 0 | 0 | 0 | 0 | 0 | 0 | 0 | 0 | 0 | 0 | 0 | 0 |
| 299 | 7 | 2 | 0 | 0 | 0 | 0 | 0 | 0 | 2 | 0 | 0 | 0 | 0 |
| 300 | 7 | 0 | 0 | 0 | 0 | 0 | 0 | 0 | 0 | 1 | 0 | 0 | 1 |

**Supplementary Table 2.** Details of each full-arch intraoral scan (IOS) and corresponding prediction errors (Model 5), including tooth count, dental conditions (missing tooth, prepared tooth, implant, orthodontic appliance, residual root, residual crown, and partially erupted tooth), error types (false positive, false negative, and wrong label) and errors in total. “+” and “–” indicate the presence or absence of each dental condition, respectively. All other values represent the number of teeth per scan and the count of teeth with associated errors per scan.

| **No.** | **Tooth Count** | **Dental Conditions** | | | | | | |  | **Error Types** | | | **Total Errors** |
| --- | --- | --- | --- | --- | --- | --- | --- | --- | --- | --- | --- | --- | --- |
|  |  | **Missing Tooth** | **Prepared Tooth** | **Implant** | **Orthodontic Appliance** | **Residual Root** | **Residual Crown** | **Partially Erupted Tooth** |  | **False Positive** | **False Negative** | **Wrong Labels** |  |
| 001 | 16 | - | - | - | - | - | - | - |  | 0 | 0 | 0 | 0 |
| 002 | 14 | - | - | - | - | - | - | - |  | 0 | 0 | 0 | 0 |
| 003 | 14 | - | - | - | - | - | - | + |  | 0 | 0 | 0 | 0 |
| 004 | 13 | - | - | - | - | - | - | + |  | 0 | 0 | 0 | 0 |
| 005 | 15 | - | - | - | - | - | - | - |  | 0 | 0 | 0 | 0 |
| 006 | 16 | - | - | - | - | - | - | - |  | 0 | 0 | 0 | 0 |
| 007 | 16 | - | - | - | - | - | - | - |  | 0 | 0 | 0 | 0 |
| 008 | 14 | - | - | - | - | - | - | - |  | 0 | 0 | 0 | 0 |
| 009 | 14 | - | - | - | - | - | - | - |  | 0 | 0 | 0 | 0 |
| 010 | 12 | + | - | - | - | - | - | - |  | 1 | 0 | 0 | 1 |
| 011 | 15 | - | - | - | - | - | - | - |  | 1 | 1 | 0 | 2 |
| 012 | 15 | - | - | - | - | - | - | - |  | 0 | 0 | 0 | 0 |
| 013 | 10 | + | - | - | - | - | - | - |  | 0 | 0 | 0 | 0 |
| 014 | 15 | - | - | - | - | - | - | - |  | 0 | 0 | 0 | 0 |
| 015 | 11 | + | - | - | - | - | - | - |  | 0 | 0 | 0 | 0 |
| 016 | 13 | - | - | - | - | + | - | - |  | 0 | 0 | 0 | 0 |
| 017 | 11 | + | + | - | - | - | - | - |  | 0 | 0 | 0 | 0 |
| 018 | 15 | - | - | - | - | - | - | - |  | 0 | 0 | 0 | 0 |
| 019 | 14 | - | - | - | - | - | - | - |  | 0 | 0 | 0 | 0 |
| 020 | 14 | - | - | - | - | - | - | - |  | 0 | 0 | 0 | 0 |
| 021 | 14 | - | - | - | + | - | - | + |  | 0 | 0 | 0 | 0 |
| 022 | 12 | - | - | - | - | - | - | + |  | 0 | 0 | 0 | 0 |
| 023 | 14 | + | - | - | + | + | - | - |  | 0 | 0 | 0 | 0 |
| 024 | 11 | + | - | + | - | + | - | - |  | 0 | 1 | 0 | 1 |
| 025 | 14 | - | - | - | - | - | - | - |  | 0 | 0 | 0 | 0 |
| 026 | 11 | + | - | - | - | - | - | - |  | 0 | 0 | 0 | 0 |
| 027 | 14 | - | + | - | - | - | - | - |  | 0 | 1 | 0 | 1 |
| 028 | 14 | - | - | - | - | - | - | - |  | 0 | 0 | 0 | 0 |
| 029 | 13 | - | + | - | - | - | - | - |  | 0 | 0 | 0 | 0 |
| 030 | 14 | - | - | - | - | - | - | - |  | 0 | 0 | 0 | 0 |
| 031 | 12 | - | - | - | - | - | - | - |  | 0 | 0 | 0 | 0 |
| 032 | 11 | + | - | - | - | - | - | - |  | 0 | 0 | 0 | 0 |
| 033 | 12 | + | - | - | - | - | - | - |  | 0 | 0 | 0 | 0 |
| 034 | 14 | - | - | - | - | - | - | - |  | 0 | 0 | 0 | 0 |
| 035 | 14 | - | - | - | - | - | - | - |  | 0 | 1 | 0 | 1 |
| 036 | 11 | + | - | - | - | - | - | - |  | 0 | 0 | 0 | 0 |
| 037 | 13 | + | + | - | - | - | - | - |  | 2 | 0 | 0 | 2 |
| 038 | 13 | - | - | - | - | - | - | - |  | 0 | 0 | 0 | 0 |
| 039 | 13 | + | + | - | - | - | - | - |  | 0 | 0 | 0 | 0 |
| 040 | 13 | - | - | - | - | - | - | - |  | 0 | 0 | 0 | 0 |
| 041 | 11 | + | + | - | - | - | - | - |  | 0 | 0 | 0 | 0 |
| 042 | 10 | + | - | - | - | - | - | - |  | 0 | 0 | 0 | 0 |
| 043 | 15 | - | + | - | - | - | - | - |  | 0 | 0 | 0 | 0 |
| 044 | 13 | + | + | - | - | + | - | - |  | 0 | 0 | 0 | 0 |
| 045 | 14 | - | - | - | - | - | - | - |  | 0 | 0 | 0 | 0 |
| 046 | 14 | - | - | - | - | - | - | - |  | 0 | 0 | 0 | 0 |
| 047 | 16 | - | - | - | - | - | - | - |  | 1 | 0 | 0 | 1 |
| 048 | 16 | - | + | - | - | - | - | - |  | 1 | 0 | 0 | 1 |
| 049 | 15 | - | - | - | - | - | - | - |  | 0 | 0 | 0 | 0 |
| 050 | 13 | + | + | - | - | - | - | - |  | 0 | 0 | 0 | 0 |
| 051 | 14 | - | - | - | - | - | - | - |  | 1 | 0 | 0 | 1 |
| 052 | 15 | - | - | - | - | - | - | - |  | 0 | 0 | 0 | 0 |
| 053 | 14 | - | - | - | - | - | - | - |  | 0 | 0 | 0 | 0 |
| 054 | 15 | - | - | - | - | - | + | + |  | 0 | 0 | 0 | 0 |
| 055 | 14 | - | - | - | - | - | - | - |  | 0 | 0 | 0 | 0 |
| 056 | 14 | - | - | - | - | - | - | - |  | 0 | 0 | 0 | 0 |
| 057 | 15 | - | - | - | - | - | - | - |  | 0 | 1 | 0 | 1 |
| 058 | 11 | + | + | - | - | - | + | - |  | 0 | 0 | 0 | 0 |
| 059 | 14 | - | + | - | - | - | - | - |  | 1 | 0 | 3 | 4 |
| 060 | 16 | - | - | - | - | - | - | - |  | 0 | 0 | 1 | 1 |
| 061 | 14 | + | + | - | - | - | - | - |  | 0 | 0 | 0 | 0 |
| 062 | 13 | + | + | - | - | - | - | - |  | 0 | 0 | 0 | 0 |
| 063 | 14 | - | - | - | - | - | - | - |  | 0 | 0 | 1 | 1 |
| 064 | 14 | - | - | - | - | - | - | - |  | 0 | 0 | 0 | 0 |
| 065 | 14 | - | - | - | - | - | - | - |  | 0 | 0 | 0 | 0 |
| 066 | 14 | - | + | - | - | - | - | - |  | 0 | 0 | 0 | 0 |
| 067 | 12 | + | + | - | - | - | - | - |  | 0 | 0 | 0 | 0 |
| 068 | 12 | - | - | - | - | - | - | - |  | 0 | 0 | 0 | 0 |
| 069 | 14 | - | - | - | - | - | - | - |  | 1 | 0 | 4 | 5 |
| 070 | 14 | - | - | - | - | - | - | + |  | 0 | 0 | 6 | 6 |
| 071 | 12 | - | - | - | - | - | - | - |  | 0 | 0 | 0 | 0 |
| 072 | 12 | - | - | - | - | - | - | - |  | 0 | 0 | 0 | 0 |
| 073 | 11 | + | - | - | - | - | - | - |  | 0 | 0 | 0 | 0 |
| 074 | 14 | - | - | + | - | - | - | - |  | 0 | 0 | 0 | 0 |
| 075 | 14 | - | - | - | + | - | - | - |  | 0 | 0 | 0 | 0 |
| 076 | 14 | - | - | - | + | - | - | - |  | 0 | 0 | 0 | 0 |
| 077 | 13 | + | + | - | - | + | - | - |  | 1 | 0 | 0 | 1 |
| 078 | 16 | - | + | - | - | - | - | - |  | 0 | 0 | 0 | 0 |
| 079 | 14 | + | - | - | - | - | - | - |  | 0 | 0 | 0 | 0 |
| 080 | 14 | + | + | - | - | - | - | - |  | 0 | 0 | 0 | 0 |
| 081 | 14 | - | - | - | - | - | - | - |  | 0 | 0 | 2 | 2 |
| 082 | 14 | - | - | - | - | - | - | - |  | 0 | 0 | 0 | 0 |
| 083 | 14 | - | - | - | - | - | - | - |  | 0 | 0 | 0 | 0 |
| 084 | 11 | + | - | - | - | - | - | - |  | 0 | 0 | 0 | 0 |
| 085 | 10 | + | - | - | - | - | - | - |  | 0 | 0 | 0 | 0 |
| 086 | 14 | - | - | - | - | - | - | - |  | 0 | 0 | 0 | 0 |
| 087 | 14 | - | - | - | - | - | - | - |  | 0 | 0 | 4 | 4 |
| 088 | 14 | - | - | - | - | - | - | - |  | 1 | 0 | 0 | 1 |
| 089 | 14 | - | + | + | - | - | - | - |  | 0 | 0 | 2 | 2 |
| 090 | 12 | + | + | - | - | - | - | - |  | 0 | 0 | 0 | 0 |
| 091 | 11 | + | - | - | - | - | - | - |  | 1 | 0 | 0 | 1 |
| 092 | 14 | + | - | + | - | + | - | - |  | 0 | 0 | 0 | 0 |
| 093 | 15 | - | - | - | - | - | - | - |  | 0 | 0 | 0 | 0 |
| 094 | 15 | - | - | - | - | - | - | - |  | 0 | 0 | 0 | 0 |
| 095 | 15 | - | - | - | - | - | - | + |  | 0 | 0 | 0 | 0 |
| 096 | 16 | - | - | - | - | - | - | + |  | 0 | 0 | 0 | 0 |
| 097 | 14 | + | + | - | - | - | - | - |  | 0 | 0 | 0 | 0 |
| 098 | 14 | - | + | - | - | - | - | - |  | 0 | 0 | 0 | 0 |
| 099 | 14 | - | - | - | - | - | - | - |  | 0 | 0 | 0 | 0 |
| 100 | 14 | - | - | - | - | - | - | - |  | 0 | 0 | 0 | 0 |
| 101 | 12 | + | - | - | - | - | - | - |  | 0 | 0 | 0 | 0 |
| 102 | 15 | + | - | - | - | - | - | - |  | 0 | 0 | 0 | 0 |
| 103 | 12 | + | - | - | - | - | - | - |  | 0 | 0 | 0 | 0 |
| 104 | 15 | - | - | - | - | - | - | - |  | 0 | 0 | 0 | 0 |
| 105 | 14 | - | - | - | - | - | - | - |  | 0 | 0 | 0 | 0 |
| 106 | 14 | - | - | - | - | - | - | - |  | 0 | 0 | 0 | 0 |
| 107 | 13 | + | - | - | - | - | - | - |  | 0 | 0 | 0 | 0 |
| 108 | 13 | + | + | - | - | + | - | - |  | 0 | 0 | 0 | 0 |
| 109 | 12 | + | - | - | - | - | - | - |  | 0 | 0 | 0 | 0 |
| 110 | 14 | - | + | - | - | + | - | - |  | 0 | 0 | 0 | 0 |
| 111 | 14 | - | - | - | - | - | - | - |  | 0 | 0 | 0 | 0 |
| 112 | 14 | - | + | - | - | - | - | - |  | 0 | 0 | 0 | 0 |
| 113 | 15 | - | - | - | - | - | - | - |  | 1 | 0 | 0 | 1 |
| 114 | 13 | + | - | - | - | + | - | - |  | 0 | 0 | 0 | 0 |
| 115 | 15 | - | - | - | - | - | - | - |  | 0 | 0 | 0 | 0 |
| 116 | 16 | - | - | - | - | - | - | - |  | 0 | 0 | 0 | 0 |
| 117 | 14 | - | - | - | - | - | - | - |  | 0 | 0 | 0 | 0 |
| 118 | 14 | - | - | - | - | - | - | - |  | 0 | 0 | 0 | 0 |
| 119 | 14 | - | - | - | - | - | - | - |  | 0 | 0 | 0 | 0 |
| 120 | 13 | - | - | - | - | - | - | + |  | 0 | 0 | 0 | 0 |
| 121 | 14 | + | - | - | - | - | - | + |  | 0 | 0 | 0 | 0 |
| 122 | 12 | + | + | - | - | - | - | - |  | 0 | 0 | 0 | 0 |
| 123 | 11 | + | + | - | - | - | - | - |  | 0 | 0 | 0 | 0 |
| 124 | 15 | - | - | - | - | - | - | - |  | 0 | 0 | 0 | 0 |
| 125 | 14 | - | - | - | + | - | - | - |  | 0 | 0 | 0 | 0 |
| 126 | 14 | - | - | - | + | - | + | - |  | 0 | 0 | 0 | 0 |
| 127 | 10 | + | - | - | - | - | - | - |  | 0 | 0 | 0 | 0 |
| 128 | 14 | - | - | - | - | - | - | - |  | 0 | 0 | 0 | 0 |
| 129 | 14 | - | - | - | + | - | - | - |  | 0 | 0 | 0 | 0 |
| 130 | 14 | - | - | - | + | - | - | - |  | 0 | 0 | 0 | 0 |
| 131 | 14 | - | - | - | - | - | - | - |  | 0 | 0 | 0 | 0 |
| 132 | 14 | - | - | - | - | - | - | - |  | 0 | 0 | 0 | 0 |
| 133 | 13 | + | - | - | - | - | - | - |  | 0 | 0 | 0 | 0 |
| 134 | 13 | + | - | - | - | - | - | - |  | 0 | 0 | 0 | 0 |
| 135 | 14 | - | - | - | - | - | - | - |  | 0 | 0 | 2 | 2 |
| 136 | 12 | - | - | - | - | - | - | - |  | 0 | 0 | 0 | 0 |
| 137 | 16 | - | - | - | - | - | - | - |  | 0 | 0 | 0 | 0 |
| 138 | 14 | - | - | - | - | - | - | - |  | 0 | 0 | 0 | 0 |
| 139 | 14 | - | - | - | - | - | - | - |  | 0 | 0 | 0 | 0 |
| 140 | 14 | - | - | - | - | - | - | + |  | 0 | 0 | 0 | 0 |
| 141 | 15 | - | + | - | - | - | - | - |  | 0 | 0 | 0 | 0 |
| 142 | 14 | - | - | - | - | - | - | - |  | 0 | 0 | 3 | 3 |
| 143 | 13 | + | - | - | - | - | - | - |  | 1 | 1 | 0 | 2 |
| 144 | 14 | - | - | - | - | - | - | - |  | 0 | 1 | 0 | 1 |
| 145 | 14 | + | - | - | - | - | - | - |  | 0 | 0 | 0 | 0 |
| 146 | 13 | + | - | - | - | - | - | - |  | 1 | 0 | 0 | 1 |
| 147 | 13 | - | - | - | - | - | - | - |  | 0 | 0 | 0 | 0 |
| 148 | 12 | - | - | - | - | - | - | - |  | 0 | 0 | 0 | 0 |
| 149 | 14 | - | - | - | - | - | - | - |  | 1 | 0 | 0 | 1 |
| 150 | 14 | - | - | - | - | - | - | - |  | 1 | 0 | 0 | 1 |
| 151 | 13 | + | - | - | - | - | - | - |  | 0 | 1 | 0 | 1 |
| 152 | 14 | - | - | - | - | - | - | - |  | 0 | 0 | 0 | 0 |
| 153 | 15 | + | + | - | - | - | - | - |  | 1 | 0 | 0 | 1 |
| 154 | 15 | - | - | - | - | - | - | - |  | 0 | 0 | 0 | 0 |
| 155 | 15 | - | - | - | - | - | - | - |  | 0 | 0 | 0 | 0 |
| 156 | 16 | - | - | - | - | - | - | - |  | 0 | 0 | 0 | 0 |
| 157 | 14 | - | + | - | - | - | - | - |  | 0 | 0 | 1 | 1 |
| 158 | 12 | + | - | - | - | - | - | - |  | 0 | 0 | 0 | 0 |
| 159 | 14 | - | - | - | - | - | - | - |  | 0 | 0 | 0 | 0 |
| 160 | 14 | - | - | - | - | - | - | - |  | 0 | 1 | 0 | 1 |
| 161 | 14 | - | + | - | - | + | - | - |  | 0 | 0 | 0 | 0 |
| 162 | 14 | - | + | - | - | - | - | - |  | 0 | 0 | 0 | 0 |
| 163 | 15 | - | - | - | - | - | - | - |  | 0 | 2 | 4 | 6 |
| 164 | 14 | - | + | - | - | - | - | - |  | 0 | 0 | 0 | 0 |
| 165 | 14 | - | + | - | - | - | - | - |  | 0 | 0 | 0 | 0 |
| 166 | 16 | - | - | - | + | - | - | - |  | 0 | 0 | 0 | 0 |
| 167 | 15 | - | - | - | - | - | - | - |  | 0 | 0 | 0 | 0 |
| 168 | 12 | + | - | - | - | - | - | - |  | 0 | 0 | 0 | 0 |
| 169 | 13 | + | - | - | - | - | - | - |  | 1 | 0 | 0 | 1 |
| 170 | 15 | - | - | + | - | + | - | - |  | 0 | 0 | 0 | 0 |
| 171 | 13 | + | + | - | - | - | - | - |  | 0 | 0 | 6 | 6 |
| 172 | 13 | + | - | - | - | - | - | - |  | 0 | 0 | 0 | 0 |
| 173 | 14 | - | + | - | - | - | - | - |  | 0 | 0 | 1 | 1 |
| 174 | 15 | - | + | - | - | - | - | - |  | 0 | 0 | 0 | 0 |
| 175 | 14 | - | - | - | - | - | - | - |  | 0 | 0 | 0 | 0 |
| 176 | 14 | + | - | - | - | - | - | - |  | 0 | 0 | 0 | 0 |
| 177 | 16 | - | - | - | - | - | - | - |  | 0 | 0 | 0 | 0 |
| 178 | 15 | - | + | - | - | - | - | - |  | 0 | 0 | 0 | 0 |
| 179 | 13 | + | + | - | - | + | - | - |  | 1 | 0 | 0 | 1 |
| 180 | 14 | + | - | - | - | + | + | - |  | 0 | 1 | 0 | 1 |
| 181 | 12 | + | - | - | - | - | - | - |  | 0 | 0 | 0 | 0 |
| 182 | 12 | + | - | - | - | - | - | - |  | 0 | 0 | 0 | 0 |
| 183 | 11 | + | - | - | - | - | - | - |  | 0 | 0 | 0 | 0 |
| 184 | 14 | + | - | - | - | - | - | - |  | 0 | 0 | 0 | 0 |
| 185 | 16 | - | - | - | - | - | - | - |  | 0 | 0 | 0 | 0 |
| 186 | 16 | - | - | - | - | - | - | - |  | 0 | 0 | 0 | 0 |
| 187 | 14 | + | - | - | - | - | - | - |  | 0 | 0 | 0 | 0 |
| 188 | 15 | - | - | - | - | - | - | - |  | 0 | 1 | 0 | 1 |
| 189 | 15 | - | - | - | - | - | - | - |  | 0 | 1 | 0 | 1 |
| 190 | 14 | - | - | - | - | - | - | - |  | 0 | 0 | 0 | 0 |
| 191 | 14 | - | - | - | - | - | - | - |  | 0 | 0 | 3 | 3 |
| 192 | 14 | - | - | - | - | - | - | - |  | 0 | 0 | 0 | 0 |
| 193 | 14 | - | - | - | + | - | - | - |  | 2 | 1 | 0 | 3 |
| 194 | 14 | - | - | - | + | - | - | - |  | 0 | 0 | 0 | 0 |
| 195 | 16 | - | - | - | - | - | - | + |  | 0 | 0 | 0 | 0 |
| 196 | 16 | - | - | - | - | - | - | - |  | 0 | 0 | 0 | 0 |
| 197 | 14 | - | - | - | - | - | - | - |  | 0 | 0 | 0 | 0 |
| 198 | 16 | - | - | - | - | - | - | - |  | 0 | 0 | 0 | 0 |
| 199 | 13 | + | + | - | - | - | - | - |  | 0 | 0 | 0 | 0 |
| 200 | 16 | - | - | - | - | - | - | - |  | 0 | 0 | 0 | 0 |
| 201 | 10 | + | - | - | - | - | - | - |  | 0 | 0 | 0 | 0 |
| 202 | 14 | - | - | - | - | - | - | - |  | 0 | 0 | 0 | 0 |
| 203 | 14 | - | - | - | - | - | + | - |  | 0 | 0 | 0 | 0 |
| 204 | 15 | - | - | - | - | + | - | + |  | 0 | 0 | 0 | 0 |
| 205 | 14 | - | - | - | - | - | - | - |  | 0 | 0 | 0 | 0 |
| 206 | 14 | - | - | - | - | - | - | - |  | 0 | 1 | 0 | 1 |
| 207 | 14 | - | - | - | - | - | - | - |  | 0 | 0 | 0 | 0 |
| 208 | 14 | - | - | - | - | - | - | - |  | 0 | 0 | 0 | 0 |
| 209 | 15 | - | - | - | - | + | - | + |  | 0 | 0 | 0 | 0 |
| 210 | 15 | - | - | - | - | + | - | - |  | 0 | 0 | 0 | 0 |
| 211 | 14 | - | - | - | - | - | - | - |  | 0 | 0 | 0 | 0 |
| 212 | 14 | - | - | - | - | - | - | - |  | 0 | 0 | 0 | 0 |
| 213 | 15 | - | - | - | - | - | - | - |  | 0 | 0 | 0 | 0 |
| 214 | 16 | - | - | - | - | - | - | - |  | 0 | 0 | 0 | 0 |
| 215 | 15 | + | + | - | - | - | + | - |  | 0 | 0 | 0 | 0 |
| 216 | 13 | + | - | - | - | - | - | - |  | 0 | 0 | 0 | 0 |
| 217 | 8 | + | - | - | - | - | - | - |  | 0 | 0 | 0 | 0 |
| 218 | 11 | - | - | - | - | - | - | - |  | 0 | 0 | 0 | 0 |
| 219 | 15 | - | - | - | - | - | - | - |  | 0 | 0 | 0 | 0 |
| 220 | 14 | - | - | - | - | - | - | - |  | 0 | 0 | 0 | 0 |
| 221 | 11 | + | - | - | - | - | - | - |  | 0 | 0 | 0 | 0 |
| 222 | 13 | - | - | - | - | - | - | - |  | 0 | 0 | 0 | 0 |
| 223 | 14 | - | - | - | - | - | - | - |  | 0 | 0 | 0 | 0 |
| 224 | 14 | - | - | - | - | - | - | - |  | 0 | 0 | 0 | 0 |
| 225 | 14 | - | - | - | - | - | - | - |  | 0 | 0 | 0 | 0 |
| 226 | 14 | - | - | - | - | - | - | - |  | 0 | 0 | 0 | 0 |
| 227 | 14 | - | - | - | - | - | - | - |  | 0 | 0 | 0 | 0 |
| 228 | 14 | - | - | - | - | - | - | - |  | 0 | 0 | 0 | 0 |
| 229 | 16 | - | - | - | - | - | - | + |  | 0 | 0 | 0 | 0 |
| 230 | 15 | - | - | - | - | - | - | - |  | 0 | 0 | 0 | 0 |
| 231 | 10 | + | - | - | - | - | - | - |  | 1 | 0 | 0 | 1 |
| 232 | 13 | - | - | - | - | - | - | - |  | 1 | 1 | 0 | 2 |
| 233 | 14 | - | - | - | - | - | - | - |  | 0 | 0 | 0 | 0 |
| 234 | 14 | - | - | - | - | - | - | - |  | 0 | 0 | 0 | 0 |
| 235 | 12 | + | - | - | - | - | - | - |  | 1 | 0 | 0 | 1 |
| 236 | 12 | + | - | - | - | - | - | - |  | 0 | 0 | 0 | 0 |
| 237 | 14 | + | - | - | - | - | - | - |  | 0 | 0 | 0 | 0 |
| 238 | 15 | - | - | - | - | - | - | - |  | 0 | 0 | 0 | 0 |
| 239 | 15 | + | - | - | - | - | - | - |  | 0 | 0 | 0 | 0 |
| 240 | 15 | - | - | - | - | - | - | - |  | 0 | 0 | 0 | 0 |
| 241 | 14 | - | - | - | - | - | - | - |  | 0 | 0 | 0 | 0 |
| 242 | 14 | - | - | - | - | - | - | - |  | 1 | 0 | 0 | 1 |
| 243 | 14 | - | - | - | - | - | - | - |  | 0 | 0 | 0 | 0 |
| 244 | 14 | - | - | - | - | - | - | - |  | 0 | 0 | 3 | 3 |
| 245 | 16 | - | - | - | - | - | - | - |  | 0 | 0 | 0 | 0 |
| 246 | 13 | + | - | - | - | - | - | - |  | 0 | 0 | 0 | 0 |
| 247 | 13 | + | - | - | - | - | - | - |  | 0 | 0 | 0 | 0 |
| 248 | 14 | - | - | - | - | - | - | - |  | 0 | 0 | 0 | 0 |
| 249 | 14 | - | - | - | - | - | - | + |  | 0 | 0 | 0 | 0 |
| 250 | 13 | + | - | - | - | - | - | - |  | 0 | 0 | 0 | 0 |
| 251 | 14 | - | - | - | - | - | - | + |  | 1 | 0 | 0 | 1 |
| 252 | 13 | + | - | - | - | - | - | - |  | 0 | 1 | 0 | 1 |
| 253 | 13 | + | - | - | - | - | - | - |  | 0 | 0 | 0 | 0 |
| 254 | 12 | + | - | - | - | - | - | - |  | 0 | 0 | 0 | 0 |
| 255 | 12 | + | - | - | - | - | - | - |  | 0 | 0 | 4 | 4 |
| 256 | 12 | - | - | - | - | - | - | - |  | 0 | 0 | 0 | 0 |
| 257 | 12 | + | - | - | + | - | + | - |  | 0 | 0 | 0 | 0 |
| 258 | 14 | - | - | - | + | - | - | - |  | 0 | 0 | 0 | 0 |
| 259 | 15 | - | - | - | - | - | - | + |  | 1 | 0 | 0 | 1 |
| 260 | 14 | - | - | - | - | - | - | - |  | 2 | 0 | 0 | 2 |
| 261 | 14 | - | - | - | - | - | - | + |  | 1 | 0 | 0 | 1 |
| 262 | 14 | - | - | - | - | - | - | - |  | 0 | 0 | 0 | 0 |
| 263 | 14 | - | - | - | - | - | - | - |  | 0 | 1 | 5 | 6 |
| 264 | 14 | - | - | - | - | - | - | - |  | 0 | 0 | 0 | 0 |
| 265 | 12 | - | - | - | - | - | - | - |  | 0 | 0 | 0 | 0 |
| 266 | 12 | - | - | - | - | - | - | - |  | 0 | 0 | 0 | 0 |
| 267 | 14 | - | - | - | - | - | - | - |  | 1 | 1 | 1 | 3 |
| 268 | 12 | - | - | - | - | - | - | - |  | 1 | 1 | 0 | 2 |
| 269 | 14 | - | - | - | - | - | - | - |  | 0 | 0 | 0 | 0 |
| 270 | 13 | + | - | - | - | - | - | + |  | 0 | 0 | 0 | 0 |
| 271 | 14 | - | - | - | - | - | - | - |  | 0 | 0 | 0 | 0 |
| 272 | 14 | - | - | - | - | - | - | - |  | 0 | 0 | 0 | 0 |
| 273 | 15 | - | - | - | - | - | - | - |  | 0 | 0 | 0 | 0 |
| 274 | 15 | - | - | - | - | - | - | - |  | 1 | 0 | 0 | 1 |
| 275 | 14 | + | - | - | - | - | - | - |  | 0 | 0 | 0 | 0 |
| 276 | 10 | + | - | - | - | - | - | - |  | 0 | 0 | 0 | 0 |
| 277 | 13 | + | - | - | - | - | - | - |  | 0 | 0 | 0 | 0 |
| 278 | 12 | + | - | - | - | - | - | - |  | 0 | 0 | 0 | 0 |
| 279 | 10 | + | + | - | - | - | - | - |  | 0 | 0 | 0 | 0 |
| 280 | 12 | + | - | - | - | - | - | - |  | 0 | 0 | 0 | 0 |
| 281 | 9 | + | - | - | - | - | - | - |  | 0 | 0 | 0 | 0 |
| 282 | 12 | + | - | - | - | - | - | - |  | 0 | 0 | 0 | 0 |
| 283 | 11 | + | - | - | - | - | - | - |  | 1 | 0 | 0 | 1 |
| 284 | 10 | + | - | - | - | + | - | - |  | 0 | 0 | 0 | 0 |
| 285 | 13 | + | - | - | - | - | - | - |  | 0 | 0 | 0 | 0 |
| 286 | 12 | + | - | - | - | - | - | - |  | 0 | 0 | 0 | 0 |
| 287 | 12 | + | - | - | - | - | - | - |  | 1 | 0 | 0 | 1 |
| 288 | 9 | + | - | - | - | - | - | - |  | 0 | 0 | 0 | 0 |
| 289 | 15 | + | - | - | - | - | - | - |  | 0 | 0 | 0 | 0 |
| 290 | 13 | + | - | - | - | - | - | - |  | 0 | 0 | 0 | 0 |
| 291 | 12 | + | - | - | - | - | - | - |  | 0 | 0 | 0 | 0 |
| 292 | 9 | + | - | - | - | + | - | - |  | 0 | 0 | 0 | 0 |
| 293 | 11 | + | - | - | - | - | - | - |  | 1 | 1 | 0 | 2 |
| 294 | 12 | + | - | - | - | - | - | - |  | 0 | 0 | 0 | 0 |
| 295 | 13 | + | - | - | - | - | - | - |  | 0 | 1 | 0 | 1 |
| 296 | 13 | + | - | - | - | - | - | - |  | 0 | 0 | 0 | 0 |
| 297 | 11 | + | - | - | - | - | - | - |  | 0 | 0 | 0 | 0 |
| 298 | 8 | + | - | - | - | + | - | - |  | 0 | 0 | 0 | 0 |
| 299 | 12 | + | - | - | - | - | - | - |  | 0 | 0 | 0 | 0 |
| 300 | 13 | + | - | - | - | - | - | - |  | 1 | 0 | 0 | 1 |
